# A framework for local-level economic evaluation to inform implementation decisions: health service interventions to prevent hospital-acquired hypoglycemia

**DOI:** 10.1017/S0266462323002775

**Published:** 2023-12-20

**Authors:** Jodi Gray, Tilenka R. Thynne, Vaughn Eaton, Brianna Reade, Rebecca Larcombe, Linda Baldacchino, Jessica Gehlert, Paul Hakendorf, Jonathan Karnon, for the Southern Adelaide Local Health Network’s (SALHN) Hypoglycaemia Clinical Working Group

**Affiliations:** 1 Flinders Health and Medical Research Institute (FHMRI), College of Medicine and Public Health, Flinders University, Bedford Park, SA, Australia; 2 Flinders Medical Centre, Southern Adelaide Local Health Network (SALHN), Bedford Park, SA, Australia; 3 College of Medicine and Public Health, Flinders University, Bedford Park, South Australia, Australia; 4 SA Pharmacy Southern Adelaide Local Health Network (SALHN), Department of Health and Wellbeing, SA Health, Government of South Australia, Bedford Park, SA, Australia

**Keywords:** decision analytic framework, local healthcare evaluation, elicitation, cost-consequence analysis, healthcare-acquired complications, hospital-acquired complications

## Abstract

**Objectives:**

Published evidence on health service interventions should inform decision-making in local health services, but primary effectiveness studies and cost-effectiveness analyses are unlikely to reflect contexts other than those in which the evaluations were undertaken. A ten-step framework was developed and applied to use published evidence as the basis for local-level economic evaluations that estimate the expected costs and effects of new service intervention options in specific local contexts.

**Methods:**

Working with a multidisciplinary group of local clinicians, the framework was applied to evaluate intervention options for preventing hospital-acquired hypoglycemia. The framework included: clinical audit and analyses of local health systems data to understand the local context and estimate baseline event rates; pragmatic literature review to identify evidence on relevant intervention options; expert elicitation to adjust published intervention effect estimates to reflect the local context; and modeling to synthesize and calibrate data derived from the disparate data sources.

**Results:**

From forty-seven studies identified in the literature review, the working group selected three interventions for evaluation. The local-level economic evaluation generated estimates of intervention costs and a range of cost, capacity and patient outcome-related consequences, which informed working group recommendations to implement two of the interventions.

**Conclusions:**

The applied framework for modeled local-level economic evaluation was valued by local stakeholders, in particular the structured, formal approach to identifying and interpreting published evidence alongside local data. Key methodological issues included the handling of alternative reported outcomes and the elicitation of the expected intervention effects in the local context.

## Background

Health service delivery models are a means of organizing the effective delivery of health care. Economic evaluation can be used to inform decisions about the design and implementation of new service delivery models to address defined priority issues. However, published evidence on the cost-effectiveness of delivery models reflects the context in which they were evaluated. The evaluation context may be very different from the context of other local health services in ways that affect the expected costs and benefits of a delivery model (1).

An alternative to using published cost-effective analyses is to undertake a modeled local-level economic evaluation (LLEE) to estimate the expected costs and outcomes of alternative delivery models if implemented in the local health service of interest. Modeled LLEEs synthesize local evidence (representing current approaches to managing the population of interest and their associated costs and consequences) with estimates of the expected costs and effects of the defined intervention options. The specification of intervention options should refer to existing evidence. Preexisting interventions (as evaluated in published evaluations) could be considered for implementation in the local context. Alternatively, the published evidence could be used to inform the development of an intervention designed to reflect the local context. Modeled LLEEs should account for likely variation in the effectiveness of the intervention options due to differences between the context in which they were evaluated and the local context in which they would be implemented.

Methods developed for hospital-based health technology assessment (hospital HTA) are structured on the premise of evaluating a single health technology, in the majority of cases this is a single medical device (2;3). Existing frameworks for local evaluation have focused on organizational structures and high-level contextual factors (1), rather than providing an applied step-by-step guide to undertaking an LLEE. The aim of this study was to develop and apply a framework for the modeled LLEE of intervention options for which externally generated effectiveness evidence is available. The framework would provide a step-by-step applied guide for those within local health service settings who are unfamiliar with economic evaluation and unclear how economic evaluation may be applied in their context to meet their needs. It adapts standard methods of economic evaluation to address the requirements and idiosyncrasies of economic evaluation in a local health service setting. The framework was applied to an LLEE of service delivery models that aimed to prevent hospital-acquired hypoglycemia in the context of the Southern Adelaide Local Health Network (SALHN).

## Methods

The SALHN catchment is located in the Adelaide metropolitan area in South Australia. It covers a resident population of 355,000 (4). SALHN manages two acute care public hospitals, Flinders Medical Centre (FMC, an 800-bed principal referral hospital) and Noarlunga Hospital (a 92-bed acute group B hospital).

A decision analytic evaluation framework was developed for modeled LLEEs that involved the following steps:

1. Define the objective of the LLEE
2. Form an evaluation working group and define the Population, Intervention, Comparator, and Outcomes (PICO)
3. Analyse local data to describe the current context
4. Pragmatic literature review of intervention options
5. Working group assessment of the intervention options
6. Preliminary modeled LLEE
7. Working group rule out intervention options
8. Elicit expected intervention effect(s) in the local context
9. Final modeled LLEE
10. Working group makes recommendations to local decision-makers

## Results

The following sections describe the processes and outputs for each of the ten LLEE steps. Extensive technical details are provided in Supplementary Files 1–4.

### Step 1. Define the objective of the LLEE

The SALHN Executive organized a workshop involving over fifty clinical staff from across the health service to review benchmarking data on hospital-acquired complications (HACs) (5) and prioritize HACs to be addressed by the organization. Preventing hypoglycemic events was identified as a priority HAC based on the capacity for improvement relative to peer hospitals, the absence of existing improvement initiatives, and the clinical importance of hypoglycemia.

### Step 2. Form the evaluation working group and define the PICO

The SALHN HACs coordinator (who has a nursing background and high levels of experience in managing quality improvement projects) formed a working group that included SALHN staff with medical, nursing, pharmacy, and data analytics backgrounds, including the chair of the SALHN Medication Safety Committee. Two members of the Flinders Health and Medical Research Institute’s (FHMRI) Health Economics and Health Services Research group were included in the working group.

The study *Population* was defined as all patients admitted to an inpatient bed at FMC (the main SALHN hospital). The *Comparator* was defined as current practice at FMC. Specific *Interventions* were not defined at this stage as potential intervention options were to be informed by a review of the literature.

The working group identified that **hypo**glycemia prevention should not increase **hyper**glycemia and that reducing the number and severity of hypoglycemia events was important even if all events could not be prevented. *Outcomes* were defined to represent the numbers of severe-hypoglycemia events (<2.2 mmol/L (<40 mg/dL)), total hypoglycemia events (<4.0 mmol/L (<72 mg/dL)) and hyperglycemia events (>15.0 mmol/L (>270 mg/dL)) based on point of care blood glucose level measurements (PoC-BGLs).

### Step 3. Analyse local data to describe the current context

SALHNs current model of care included a standardized basal-bolus insulin chart and standardized hospitalwide policies for: glycemic control, hypoglycemic event management, and referral to the endocrinology consultation team.

Using an evidence-based audit tool designed with the working group, 101 admissions for adult patients with diabetes who were coded as having a hypoglycemia HAC at FMC in the 2018–19 financial year were audited. For eight of these admissions, no hypoglycemic events were identified in the audit. Across the remaining ninety-three admissions, there were 329 separate hypoglycemic events, with two or more events experienced in fifty-nine (63 percent) admissions. Most events were treated by nursing staff only (*n* = 220, 78 percent), with ten events requiring assistance from the Medical Emergency Team (i.e., an MET call; 3.5 percent).

The audit identified that most hypoglycemic events occurred in patients who:

- Were on insulin (*n* = 277, 84 percent).
- Were under the care of a medical (*n* = 145, 44 percent) or surgical team (*n* = 117, 36 percent) with few events in the ICU (*n* = 1, 0.3 percent).
- Experienced dysglycemia in the 24 hours before the event (*n* = 238, 73 percent), specifically hyperglycemia (defined as BGL >10.0 mmol/L (180 mg/dL); *n* = 172, 53 percent), hypoglycemia (BGL <4.0 mmol/L (72 mg/dL); *n* = 119, 37 percent), or both (*n* = 63, 16 percent).

Nutrition was identified as a contributing cause for 54 percent of events (*n* = 179), particularly when patients had an unpredictable oral intake (e.g., due to reduced appetite, nausea or vomiting) (*n* = 115, 35 percent) or were fasting (*n* = 49, 15 percent). The working group identified that unpredictable oral intake was a key factor in combination with unadjusted insulin doses. An increase in insulin dose by more than 10 percent or a change in insulin type contributed to 15 percent of hypoglycemic events (*n* = 48), while insulin prescribing and administration errors led to five hypoglycemic events (1.5 percent). An increase in physical activity contributed to 8 percent of events (*n* = 25). See Table S1.1 in Supplementary File 1 for all results.

### Step 4. Pragmatic literature review of intervention options

A pragmatic literature review was undertaken that identified forty-nine publications for forty-seven studies. Full details of the review are available as a published paper (6).

The review found twenty-one studies undertaken in ICU-specific settings and twenty-six in non-ICU-specific settings (e.g., non-ICU wards only, or non-ICU wards and ICU). Nine broad intervention categories were defined: services (eight articles, six studies), role expansion (*n* = 6), education (*n* = 9), audit and feedback (*n* = 1), alerts and reminders (*n* = 3), protocol implementation methods (*n* = 1), order sets (*n* = 6), insulin charts (*n* = 1), and electronic glycemic management systems (eGMS; *n* = 14). Studies were predominantly non-randomized (*n* = 40) and few adjusted key dysglycemic outcomes for potential confounding or clustering (*n* = 4/40 non-randomized studies, *n* = 3/7 RCTs).

### Step 5. Working group assessment of the intervention options

Working group members attended one of two face-to-face meetings (lasting 2 or 3 hr), where summaries of the twenty-six non-ICU-specific studies were presented for consideration (see Supplementary File 2 for example summaries). The focus on non-ICU-specific interventions was informed by the clinical audit. The working group discussed each intervention to shortlist interventions that could reduce the occurrence of hypoglycemia within SALHN. They considered the intervention design, target population, local root causes, existing local services and resources, and the overall SALHN context including the likelihood that the intervention could be implemented.

The group selected the following three interventions for inclusion in the preliminary LLEE. Table 1 describes key study characteristics and intervention effect estimates:

1. An intervention where nurses received an automated pop-up survey in the EMR soon after a hypoglycemic event occurred. The survey asked them to identify relevant characteristics and causes of the event. It acted as a reflective intervention, as well as identifying root causes to inform targeted topics for brief educational presentations (7).
2. A virtual glycemic management service (vGMS) that generated a daily report of all inpatients with dysglycemia in the last 24 hours. Endocrinologists, certified diabetes educator pharmacists, and certified diabetes educator nurses from the service remotely reviewed the patients’ electronic medical records and added recommendations on glycemic management before morning rounds (8;9).
3. A pharmacist-led peri-operative glycemic management team (GMT) took responsibility for glycemic management in surgical patients upon request of the treating surgeon (10;11).

**Table 1.** Details of the interventions selected by the working group

Intervention	Root cause survey	Virtual glycemic management service (vGMS)	Pharmacist-led peri-operative glycemic management team (GMT)
First author	Sinha Gregory (7)	Rushakoff (8;9)	Mularski (11); Mosen (10)
Publication year	2018	2017	2012 (11), 2015 (10)
Multiple papers	N/a	Both papers report the same study and analyses	Each paper reports different analyses of the same study data
Country	USA	USA	USA
Study design	Non-randomized (pre–post)	Non-randomized (pre–post)	Non-randomized (pre–post)
Division	Medical	Hospitalwide	Surgical
Patient cohort in study	All patients in two medical units with PoC-BGLs recorded	All non-obstetric adult patients (with and without PoC-BGLs recorded)	Surgical patients admitted to the PACU with 2 + PoC-BGLs during PACU admission (Mularski) or on day 1^a^ of PACU admission (Mosen). Excluded patients admitted directly to ICU (i.e., cardiovascular and critically ill surgical patients)
Patients targeted by intervention	Those with diabetes or hyperglycemia or on insulin	Those with hypoglycemia or hyperglycemia or on an insulin pump	Those with diabetes or at risk of hyperglycemia
*Published effect estimates*			
Units (% of)	BGL measurements	Patient days	Patients
Duration of the outcome measurement	Events during the hospital admission	Events on days 1–28 of hospital admission	Events on days 1–3 of PACU admission
Severe-hypoglycemia (unadjusted RR)	NR	0.31 (95% CI: 0.15, 0.59), *p <* 0.001	Mularski: 0.67, p:NR^b^ Mosen: NR
Hypoglycemia (unadjusted RR)	0.68, *p* < 0.001	0.64 (95% CI: 0.57, 0.70), *p <* 0.001	Mularski: 0.46, p:NR^c^ Mosen: 0.38, p:NR^d^
Hypoglycemia (adjusted RR)	NR	NR	Mularski: 0.36 converted^e^ from OR: 0.34 (95% CI: 0.28, 0.40) *p <* 0.001 Mosen: 0.43 converted^e^ from OR: 0.38 (95% CI: 0.31, 0.46), *p* < 0.001
Hyperglycemia^f^ (unadjusted RR)	0.85, *p* < 0.001	0.61 (95% CI: 0.59, 0.63), *p* < 0.001	NR

a Day 1 is the day of surgery and PACU admission.

b Chi-squared test using data reported in the paper gave a RR:0.68 (95% CI: 0.40, 1.14), p:0.142.

c Chi-squared test using data reported in the paper gave a RR:0.46 (95% CI: 0.38, 0.57), *p* < 0.001.

d Paper reported a comparison of three time points (preintervention vs. year 1 vs. year 2): *p <* 0.001. RR reported here is preintervention vs. year 1, for which a chi-squared test using data reported in paper gave a RR:0.38 (95% CI: 0.32, 0.44), *p* < 0.001.

e ORs converted to RRs as described by Zhang and Yu (14).

f Defined as PoC-BGL >10.0 mmol/L (180 mg/dL) for the root cause survey and as PoC-BGL ≥12.5 mmol/L (225 mg/dL) for the vGMS.

ICU, intensive care unit; N/a, not applicable; NR, not reported; OR, odds ratio; PACU, postanesthesia care unit; PoC-BGL, point-of-care blood glucose level; RR, relative risk.

A computer-guided insulin dosing calculator was also selected (12). However, this was excluded as it did not have approval from the Therapeutic Goods Administration (TGA) for use as a medical device within Australia (13).

## Step 6. Preliminary LLEE

Extensive technical details for the LLEE and findings from sensitivity analyses are provided in Supplementary Files 3 and 4.

### Local baseline event rates

For the selected interventions the working group defined the target populations. The root cause survey with targeted education (7) and the vGMS (8;9) would be implemented for all admitted adult (≥18 yr) patients, excluding obstetric patients. The pharmacist-led peri-operative GMT (10;11) would be implemented for adult surgical patients excluding obstetric but including ICU patients.

Baseline event rates were estimated for the defined target populations at FMC during 2019. Hospitalwide (excluding obstetric patients) there were 154 inpatient admissions coded as a hypoglycemia HAC. It was estimated there were 641 admissions with hypoglycemia events and 1,732 hypoglycemia events based on the analysis and extrapolation of point-of-care blood glucose level (PoC-BGL) test results (see baseline counts in Table 2). This was consistent with the working group’s belief that coded HACs significantly underestimated hypoglycemia events. Numbers for hyperglycemia events could not be estimated with the data available at the time of the analysis.

**Table 2.** Results from the preliminary and final local-level economic evaluations (LLEE): cost-consequences analysis for the interventions remaining of interest to the working group (annual)

	Root cause survey			vGMS (with reporting criteria)			
	Preliminary LLEE	Final LLEE		Preliminary LLEE	Final LLEE		Baseline counts^a^
	Estimate	Estimate	(Range)	Estimate	Estimate	(Range)	
*Costs/time resources*							
Additional salary costs (AU$)		5,275 once + 2,190 per cycle^b^			82,000^c^		
Additional staff time (hours)		88 once + 44 per cycle^b^			1,185^c^		
*Effects*							
Reduction in PoC-BGLs with:							
Severe hypoglycemia	48	22	(15–30)	75	74	(37–75)	150
Total hypoglycemia	554	259	(173–346)	619	409	(310–514)	1732
Reduction in admissions with:							
Multiple hypoglycemia events	89	39	(26–53)	98	56	(43–76)	330
Severe hypoglycemia events	34	15	(10–21)	52	52	(23–52)	125
Any hypoglycemia event	115	49	(32–67)	55	32	(24–43)	641
Coded hypoglycemia HACs^d^	27	11	(7–16)	13	7	(5–10)	154
*Savings*							
Reductions in:							
HAC financial penalty (AU$)		12,452	(7,924–18,112)		7,924	(5,660–11,320)	174,328
Occupied bed days (days)^e^		147	(96–201)		96	(72–129)	1,923
Occupied bed days cost (AU$)^e^		213,150	(139,200–291,450)		139,200	(104,400–187,050)	2,788,350
Nursing time to treat hypoglycemia events (hours)		29	(19–39)		59	(38–68)	194

*Note*: The preliminary local-level economic evaluation (LLEE) applied the published intervention effects (relative risks (RR)). The final LLEE applied the elicited, locally adjusted intervention effects. In the final LLEE effects and savings estimates were calculated using the most realistic effect estimates provided during elicitation, with the most pessimistic and most optimistic estimates used to specify a plausible range. Reported values are average reductions over 5,000 bootstraps. For the vGMS intervention criteria of two or more prior hyperglycemia events (>15.0 mmol/L (>270 mg/dL)) or one or more prior hypoglycemia events (<4.0 mmol/L (<72 mg/dL)) were used to select the patients included in the daily report for vGMS review. This analysis limited the effects of the vGMS intervention to this patient cohort.

a Estimated total baseline numbers for the analysis cohort.

b Time includes 88 hours as a once off to develop and test the survey in the EMR, and an additional 44 hours per survey cycle comprising 25 hours of nursing time to complete the survey and 19 hours of implementation and education activities. This time would be covered by existing staffing levels, with no additional salary costs required (costs provided for reference only).

c Includes an additional 0.2 FTE (full time equivalent; equal to 38 hours per week) each for an endocrinology registrar, certified diabetes educator (CDE) nurse and CDE pharmacist. This would require additional funding to employ additional staff (additional time is provided as a reference only).

d HACs were calculated in each bootstrap run as 24.0 percent of total hypoglycemic patients. This percentage was derived from FMC baseline data.

e Occupied bed-days associated with the prevention of hypoglycemic events (16).

HAC, hospital-acquired complication.

### Modeling intervention effects on hypoglycemia

A local data set was generated that described the numbers of severe-hypoglycemia and non-severe hypoglycemia events experienced during each eligible hospital admission. The modeling approach involved the application of relative risks (RRs) representing intervention effects on severe- and non-severe hypoglycemia events. For each hypoglycemic event experienced by an eligible patient, a random number between zero and one was generated. If the sampled value was greater than the relevant RR, the event was considered to have been prevented.

The estimation of intervention effects was complicated as each intervention study reported a different outcome measure:

- PoC-BGL measurements in the hypoglycemic range (7).
- Patient-days with one or more hypoglycemic events (8;9).
- Patients experiencing one or more hypoglycemic events during their admission (10;11).

**Figure 1.**
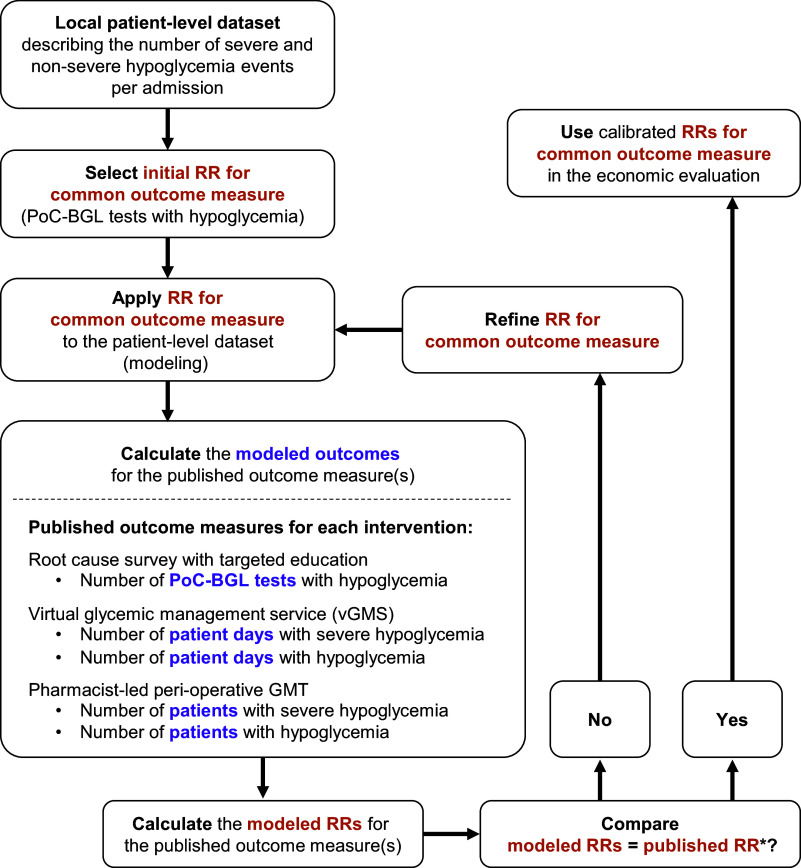
Calibrating the relative risk (RR) for a common outcome measure to enable comparison of the three interventions. The evaluations published for each intervention reported different outcome measures. To compare the three interventions required using relative risks (RRs) for a single, common outcome measure in the economic modeling. The number of PoC-BGL tests with hypoglycemia was selected as the common outcome measure. For each intervention, calibration involved selecting an initial value for the RR for the common outcome measure and applying this in the model. The modeled RR for the published outcome measure was then calculated and compared to the published RR* for the published outcome measure. The RR for the common outcome measure was refined until the modeled RR and published RR* matched. This ensured that the RR for the common outcome measure accurately represented the magnitude of the published RR*. Once calibrated, the RR for the common outcome measure was used in the economic evaluation. GMT: glycemic management team. PoC-BGL: point-of-care blood glucose level. RR: relative risk. *Published RRs in preliminary local-level economic evaluation (LLEE), elicited RRs in final LLEE.

The intervention effects reported as PoC-BGL measurements in the hypoglycemic range could be directly applied to the local data set (7). However, the two other outcome measures could not be directly applied to the local data set (8-11). Calibration was used to translate these two published interventions’ effects into effects on a common outcome measure (i.e., into effects on PoC-BGL measurements in the hypoglycemic range) which could be applied to the local data set. The calibration steps are described in Figure 1. They were as follows:

1. Apply RRs for the common outcome measure for both severe and non-severe hypoglycemia events to eligible events in the local data set.
2. Summarize the hypoglycemia outcomes in the local data set.
3. Estimate the RR for the outcome measure reported in the intervention study, for example, patient-days with one or more hypoglycemia events (termed “modeled RRs”).
4. Compare the modeled RRs (from Step 3) with the published RRs for the outcome measure reported in the intervention study.
5. Refine the RRs for the common outcome measure for both severe and non-severe hypoglycemia events (used in Step 1) and repeat Steps 1 to 4 until the predicted and published RRs converge (in Step 4).

To stabilize the outputs, the mean outputs from 5,000 bootstrapped samples were used for each model run. Convergence was defined as an absolute difference of 0.001 or less between the modeled and published RRs.

The percentage of patients coded as having a hypoglycemia HAC was 24.0 percent of patients with a hypoglycemic event for the all patients cohort (root cause survey and vGMS analyses), and 25.5 percent for the surgical patient’s cohort (pharmacist-led GMT analyses). Percentages were derived from FMC baseline data.

Analyses for the vGMS intervention modeled intervention effects across all baseline events, and when three alternative reporting criteria were used to select patients for review by the service (with intervention effects limited to those patients). Base case reporting criteria were specified by the working group to include patients with two or more hyperglycemia events (>15.0 mmol/L (>270 mg/dL)) or one or more hypoglycemia events (<4.0 mmol/L (<72 mg/dL)) in the previous 24 hours.

### Preliminary LLEE outcomes

Selected preliminary LLEE analyses are reported in Table 2, with all base case results and sensitivity analyses reported in Table S4.5 in Supplementary File 4. The modeling predicted that the vGMS intervention (with base case reporting criteria applied) prevented all hypoglycemic events in 55 patients compared to 115 with the root cause survey intervention, and 109 to 122 with the pharmacist-led peri-operative GMT (range is due to different adjusted RRs reported by Mosen et al. (10) and Mularski et al. (11) for the same study). The vGMS had a larger incremental effect on severe-hypoglycemia events, preventing fifty-two events, more than twice the number prevented by the other interventions (thirty-four with root cause survey; twenty-one to twenty-three with pharmacist-led GMT).

### Resource use

Intervention descriptions and associated resource use are presented in Table 3. The pharmacist-led peri-operative GMT required two full-time equivalent (FTE) pharmacists (10;11), which represented significantly greater resource requirements than the other two intervention options.

**Table 3.** Resource use and additional staff costs associated with the interventions of interest to the working group

Intervention	Intervention description	Resource use	Additional staff costs
Root cause survey (7)	Automated EMR survey tool completed by nursing staff for real-time identification of hypoglycemic events and root causes. Concurrent clinical audit of hypoglycemic events by an endocrinology resident for physician perspective. Brief targeted education addressing the main cause identified (insulin titration) using a 10-min PowerPoint and a 1 page handout on insulin dosing. Future education planned on interruptions in nutrition (which ranked second as a main cause). Process engages and empowers nurses (and physicians) on hypoglycemia prevention and glycemic control.	Tool development using an initial survey of nursing staff. Nursing staff time to complete survey tool and consider implications. Analysis of survey tool data. Concurrent audit of hypoglycemia events by endocrinology resident. Development of education content and materials. Delivery of education to nurses and physicians.	N/a
Virtual glycemic management service (vGMS) (8;9)	Daily automated report of potentially “at risk” patients is generated via the EMR. At risk is defined in the study as having had one or more BGLs <70 mg/dL (3.9 mmol/L) or two or more BGLs ≥225 mg/dL (12.5 mmol/L) in the last 24 hr, or prescribed an insulin pump. Report is reviewed by vGMS team prior to morning rounds. Notes added to the patients’ EMR suggesting changes to glycemic management if required.	Review of ward staff competency on insulin knowledge and regimens. vGMS team of endocrinologist, nurse CDE, and pharmacist CDE. In 540-bed hospital it takes 20–40 mins per day for the team to review the daily report and make an average of 5.3 EMR notes per day Led to four additional formal endocrinology consultations per month. MO time to consider vGMS notes and insulin changes during morning rounds.	0.2 FTE registrar: AU$40,000 0.2 FTE RN3: AU$21,000 0.2 FTE AHP3: AU$21,000 (all include oncosts)
Pharmacist-led peri-operative glycemic management team (GMT) (10;11)	GMT pharmacists provide comprehensive inpatient glycemic management (including daily review and discharge planning) for surgical patients in need of peri-operative glycemic control. Physicians order the service via the EMR (“Glucose management per pharmacy protocol”).	Adaptation / development of GMT protocol. GMT pharmacist training. In a 300-bed hospital the team manages twenty to thirty-five patients per day. This requires two FTE pharmacists drawn from a pool of thirteen trained pharmacists.	2 FTE pharmacists: AU$210,000 (excludes oncosts)

AHP, allied health provider; BGL, blood glucose level; CDE, certified diabetes educator; EMR, electronic medical record; FTE, full-time equivalent (38 hours per week); MO, medical officer; PoC, point-of-care; RN, registered nurse.

### Step 7. Working group rule out non-implementable interventions

After reviewing the results of the preliminary LLEE, the working group excluded the pharmacist-led GMT (10;11) from further consideration. This was due to the relatively high cost of the additional staff required to implement the intervention (two FTE pharmacists at an annual cost of AU$210,000), alongside the presence of two other, less resource-intensive interventions with larger expected aggregate effects (7-9).

### Step 8. Elicit expected intervention effects in the local context

Expert elicitation was used to adjust the published effect estimates of the two remaining interventions to better reflect their likely effectiveness if implemented in the local context. Full details of the elicitation process are reported in a separate paper (15), with the methods and findings summarized here. For each of the two interventions remaining of interest, the study and local (SALHN) contexts were examined by the clinical members of the working group. Evidence relating to three broad contextual factors was compiled from the published intervention studies, from web sites describing the characteristics of the hospitals and healthcare systems in which the studies were undertaken, and from the local SALHN data described in Steps 3 and 6. These factors were the:

- Baseline characteristics of the target populations at the study site and the local site.
- Baseline quality of care and hospital characteristics at the study site and the local site.
- Potential biases relating to the research study design and the impact of implementing the intervention in a research context.

The working group was presented with this evidence and asked to systematically assess the similarities and differences between the study and local sites. A summary of the discussion was presented back to the group, who were then asked to provide a most optimistic, a most pessimistic, and a most realistic estimate of the effectiveness of the intervention in the local context (i.e., quantified estimates of the RRs).

For both interventions, the discussion focused on describing the most likely drivers of differences in the baseline rates of hypoglycemia between the study hospitals and the local context. Data describing the status of the two study hospitals as highly ranked, major referral hospitals in the US informed an assumption that local (SALHN) patients may be less complex on average.

#### vGMS intervention

The group concluded it would be more challenging to address the highly variable causes of hypoglycemia in a lower complexity patient cohort, compared to a more complex cohort where risks could be more narrowly defined and targeted. Therefore, they expected the effectiveness of the vGMS intervention to be reduced when implemented locally. In terms of research context, this intervention was designed and evaluated by an endocrinology team within the study hospital. The working group thought this was unlikely to impact effectiveness, given a high level of buy-in from the local endocrinology team would be required before implementation would occur.

For hypoglycemia, the most realistic elicited RR was 0.76 (range: most optimistic 0.70 to most pessimistic 0.82), meaning all elicited intervention effects were less than the mean published RR of 0.64 (95 percent CI: 0.57 to 0.70). For severe-hypoglycemia, the most realistic locally adjusted RR was 0.50 (range: 0.20 to 0.75) compared to the published RR of 0.31 (95 percent CI: 0.15 to 0.59).

#### Root cause survey intervention

The intervention was implemented in only two general medical wards during the study, while FMC intended to implement hospitalwide. The working group thought broad implementation was likely to reduce engagement and buy-in from clinical staff on each ward, reducing the effectiveness of the intervention. In addition, broad implementation would affect the comparability of the patient populations. The group believed the intervention may be less effective in surgical patients, as they generally have a less stable nutritional intake pattern than medical patients and this would be harder to predict and prevent with a virtual review.

For hypoglycemia a locally adjusted RR of 0.85 (range: 0.80 to 0.90) was elicited compared to the mean published RR of 0.68 (95 percent CI: not reported). In the absence of a published effect estimate for severe-hypoglycemia, the working group expected the same intervention effects for hypoglycemia and severe-hypoglycemia.

### Step 9. Final LLEE

The methods described for the preliminary LLEE (Step 6) were repeated for the interventions that remained of interest, this time applying the locally adjusted (elicited) intervention effect parameters. Table 2 presents the results of the cost-consequence analysis, reporting the costs associated with the main resources required to implement and to maintain each intervention, as well as the expected effects on hypoglycemic events, cost savings and occupied bed days (see Table S4.5 in Supplementary File 4 for all sensitivity analyses).

#### Predicted intervention effects on hypoglycemia

The results presented in Table 2 show that the root cause survey intervention is expected to have a greater effect on coded HACs and total hypoglycemic events, but the vGMS intervention has a greater expected effect on severe hypoglycemia events and patients experiencing multiple events within a hospital admission.

**Figure 2.**
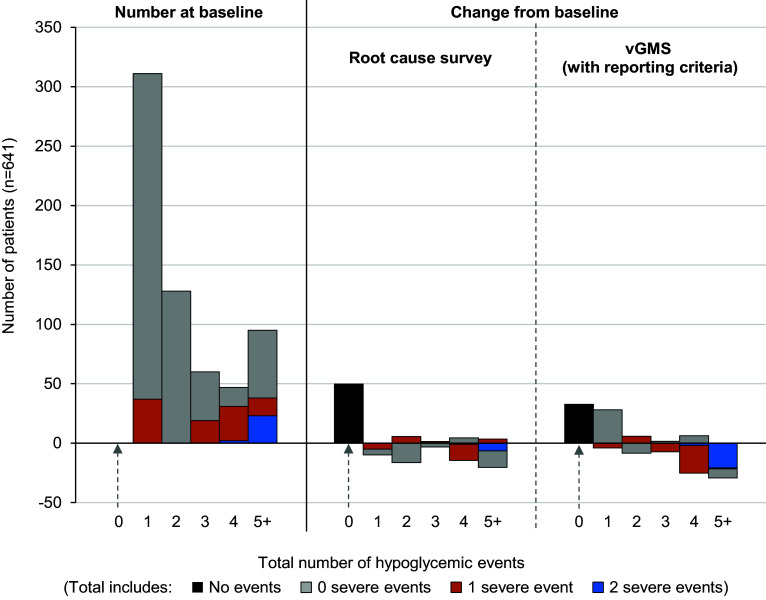
Joint distributions of severe-hypoglycemia and hypoglycemia events per patient at FMC (using elicited “most realistic” RRs for final local economic evaluation (LEE)). Estimated baseline and predicted postintervention distributions of hypoglycemic events across all patients in the FMC cohort who experienced at least one hypoglycemic event at baseline. Postintervention estimates are based on the most realistic RR elicited from the working group for each intervention. Plotted distributions are based on the average intervention effect over 5,000 bootstraps. Distribution for all patients is shown at baseline, while distributions for the interventions show the change in the number of patients experiencing that number/severity of event. For the vGMS intervention criteria of two or more prior hyperglycemia events (>15.0 mmol/L (>270 mg/dL)) or one or more prior hypoglycemia events (<4.0 mmol/L (<72 mg/dL)) were used to select the patients included in the daily report for vGMS review. The analysis limited the effects of the vGMS intervention to this patient cohort.

Figure 2 presents histograms representing the numbers of non-severe and severe hypoglycemia events experienced during individual hospital admissions in which at least one event was experienced in the baseline scenario. The figure shows the root cause survey intervention moves more people into the zero events category. The vGMS intervention increases the number of admissions with one event, but reduces admissions with three or more events or with severe events.

#### Intervention resource use and costs

The resources required to implement each intervention in the local context were estimated by the working group, and costings for the additional staff required are reported in Table 2 and Table 3. The vGMS intervention was estimated to require additional resourcing for 0.2 FTE each of an endocrinology registrar, certified diabetes educator (CDE) nurse, and CDE pharmacist (total 1,185 hr). This time would cover the daily review of reported medical records and any additional patient consultations with an endocrinologist (estimated at four per month).

Nursing time required to complete the automated pop-up surveys following each hypoglycemic event was associated with the root cause survey intervention. Applying an estimate of 1 minute to complete each survey would take twenty-five nursing hours annually if implemented hospitalwide. No additional nursing staff would be rostered on to cover this time therefore there would be no financial cost to SALHN; however, opportunity costs would need to be considered. In addition, each cycle of the survey intervention would require 19 hours of staff time for survey roll-out, survey analysis, follow-up education, and for an endocrinology resident to undertake a concurrent audit of hypoglycemia events. There would be a once-off initial time investment of 88 hours to develop and test the survey in the EMR. From the local health service’s budgetary perspective, all these resources would be covered by existing budgets and staffing levels but salary costings have been provided in Table 2 for reference.

#### HAC financial penalties

Hospital funding in Australia is reduced for any episode of admitted acute care where a coded HAC occurs (5). The average financial penalty for a hypoglycemia HAC at FMC in 2019 was estimated as AU$1,132. This was applied to the predicted numbers of coded HACs (Table 2) to give a reduction in penalty costs of AU$12,452 (range: 7,924 to 18,112) for the root cause survey intervention, and AU$7,924 (range: 5,660 to 11,320) for the vGMS intervention with the reporting criteria applied.

#### Other resource use effects

Resource use effects associated with a reduction in the number of hypoglycemic events includes reduced nursing time to manage hypoglycemia, which was estimated by the working group to be 5 minutes for a non-severe hypoglycemia event and 25 minutes for a severe-hypoglycemia event. The expected reduction in nursing time for hypoglycemia event management was 59 hours with the vGMS intervention (range: 38–68) and 29 hours (range: 19–39) with the root cause survey intervention. After subtracting the 25 hours of nursing time required to complete the root cause surveys this gave a net annual time cost saving of 4 hours (range: 14 hours saved to 6 additional hours required).

The reduced number of occupied bed-days associated with the prevention of hypoglycemic events was also estimated. Applying an estimate of 3 bed-days avoided for each admission with all hypoglycemia events prevented (16) suggests a reduction of 147 bed-days (range: 96–201) for the root cause survey and 96 bed-days (range: 72–129) for the vGMS intervention (Table 2).

Cost savings associated with this reduction in bed days were estimated by applying a median Long-Stay Outlier Per Diem price weight (0.2641, calculated across all admitted acute price weights) to the Australian national efficient price (AU$5,597 for 2021-22) to generate a cost per bed day of AU$1,450 (17;18). Applying this cost per bed-day gave a reduction of AU$213,150 (range: 139,200–291,450) for the root cause survey, and AU$139,200 (range: 104,400–187,050) for the vGMS.

### Step 10. Working group make recommendations to local decision-makers

The working group considered a range of methodological issues when interpreting the results of the final LLEE, including:

- Per patient rates of severe and non-severe hypoglycemia had to be extrapolated for FMC based on PoC-BGL data from the smaller Noarlunga hospital. This was likely to have underestimated FMC’s baseline hypoglycemia event rates, and hence underestimated the expected number of events prevented.
- Intervention effects on hypoglycemic events experienced by the same patient were assumed to be independent in the modeling. However, some correlation can be expected, which would increase the number of patients for whom all events would be prevented.
- For the vGMS intervention, broader effects (beyond the patients’ meeting the reporting criteria) were expected through improvements in clinician knowledge, insulin titration, and overall glycemic management practices.
- Greater focus on hypoglycemia prevention may improve the recording of hypoglycemia events and coding of hypoglycemia HACs, potentially increasing the HAC financial penalties in the short term.
- Reduced occupied bed days were applied only to admissions for which all hypoglycemic events were prevented (16). Reducing the number of events a patient experienced would also impact on length of stay, particularly if severe-hypoglycemic events were prevented.

The working group concluded that the root cause survey intervention was likely to provide high initial value for money, particularly if implemented in short bursts. However, its value was expected to diminish over time due to “alert fatigue” and the challenge of sustaining long-term educational interventions. The strength of the intervention was in prompting real-time reflection on individual hypoglycemic events along with education and awareness raising. However, given the local EMR had only recently been rolled-out, insertion of the pop-up survey into the EMR could be delayed by substantial wait times for the required information technology support.

The vGMS was also expected to provide value for money, despite incurring additional salary costs. This interpretation was largely due to the predicted reductions in the numbers of severe- and non-severe hypoglycemia events and the recognition that the occupied bed day savings were likely underestimated. The strength of the intervention was in providing timely, individualized, peer-to-peer feedback on insulin prescribing from an expert team with consistent messaging.

A feasibility pilot of the vGMS report found more than the allocated FTE time would be required to review all patients meeting the dysglycemia criteria, leading to the conclusion that a modified version of the intervention would need to be developed, implemented, and evaluated. For example, the vGMS team may review severe events, and provide only a generic EMR note for all other reported events.

The potential for complementary effects from the implementation of both the vGMS and the root cause survey intervention was recognized. In particular, implementing the survey could reduce the numbers of patients in the vGMS report, making it more viable to implement. Considering the low resource requirements for the root cause survey intervention, the working group recommended its implementation along with a time-limited, funded trial of a modified vGMS intervention.

## Discussion

The starting point for the study reported in this paper was the research finding that “for economic evaluation to be helpful in real-life policy decisions, it has to be placed into context” (19). This paper has described the application of a framework for the LLEE of service intervention options using effectiveness evidence generated outside of the local setting. The application of the framework to a case study economic evaluation of interventions to prevent hospital-acquired hypoglycemia was led by academic health economists working with a multidisciplinary clinical team. The framework adapts standard economic evaluation methods to address the requirements and idiosyncrasies of conducting economic evaluation in a local health service setting and to ensure the evaluations undertaken meet the needs of the local health service. Stakeholder engagement (via the working group) was critical throughout the evaluation process to define evaluation objectives; to conduct a clinical audit; to select relevant intervention options; and to interpret both the available evidence (including the elicitation of expected intervention effects) and the evaluation results in the local context. The shortlisting of feasible and acceptable intervention options was a necessary step to limit the size and scope of the economic evaluation. The evaluation of multiple healthcare delivery models is in contrast to commonly applied hospital HTA processes which primarily focus on evaluating single medical devices (2;3).

A pragmatic approach to the elicitation of expected intervention effects in the local context was used. It involved eliciting consensus effectiveness estimates from the clinical members of the working group and not seeking a larger panel of experts from across SALHN. This was appropriate given the working group comprised relevant clinical experts who had developed a good rapport whilst working on the project, enabling robust group discussions that could inform consensus decisions. Comparisons of published study contexts and the local context was a challenge due to limited published information on the baseline study contexts, but supplementation with online information on the study hospitals usefully informed the elicitation process. Expert elicitation methods have previously been used to estimate uncertain or unobserved parameters in health care decision making (20;21). For example, Yao et al. used elicitation in a preimplementation economic evaluation of a service delivery intervention to improve clinical handovers (22). After designing their intervention they elicited expected effects from experts, but this was done without reference to existing evidence on the effects of similar interventions.

Another issue concerned the use of alternative measurement units by different published intervention studies: hypoglycemic events, patient-days with hypoglycemic events, and patients experiencing hypoglycemic events. This required statistical coding to run calibration analyses to generate intervention effects using a common measure of outcome. The developed code can be adapted to address this general issue for evaluations in other clinical areas.

A limitation of the reported evaluation framework relates to the expert time and resources associated with the conduct of the evaluation (i.e., academic health economists and clinician membership of the working group). It is noted that “the time and cost required to conduct and interpret economic evaluations, and lack of expertise to evaluate quality and interpret results” have also been identified as barriers to the use of economic evaluation (23). However, transferable tools and materials were developed, including the clinical audit tool, the pragmatic literature review (6), the elicitation framework (15), and code for data analysis and economic modeling. Options for the dissemination of these resources include a consultancy model or training courses with resources developed to upskill local personnel to apply the evaluation tools and materials locally. Beyond this case study, tools and materials for the LLEE of service interventions targeted at a broad range of outcomes could be developed with accompanying support for their application in different local health service contexts.

## Conclusions

A key question is whether economic evaluation adds value to local decision-making, which can only be assessed subjectively. In the absence of the applied economic evaluation, a quality improvement project would have been initiated. In the local context, such projects refer to the literature in a less systematic manner, with more of a focus on reviewing processes of care to identify areas for improvement. Quality improvement projects tend not to consider solutions that require additional resources. Feedback from the working group was that they valued the more structured, formal approach to identifying and interpreting published evidence alongside local data, which led to recommendations for the implementation of interventions with relatively low additional resource requirements.

## Supporting information

Gray et al. supplementary material 1 Gray et al. supplementary material

Gray et al. supplementary material 2 Gray et al. supplementary material

Gray et al. supplementary material 3 Gray et al. supplementary material

Gray et al. supplementary material 4 Gray et al. supplementary material

## Supplementary material

The supplementary material for this article can be found at http://doi.org/10.1017/S0266462323002775.
