# Supplementary material for: A framework for local-level economic evaluation to inform implementation decisions: health service interventions to prevent hospital-acquired hypoglycemia
Source: Int J Technol Assess Health Care. 2023 Dec 20;39(1):e74. doi: 10.1017/S0266462323002775 (PMC11579668; doi:10.1017/S0266462323002775)
Supplement: Gray et al. supplementary material 1 — Gray et al. supplementary material [file S0266462323002775sup001.pdf]

Supplementary file 1: Findings from the medical record audit

This supplementary file presents the findings from the medical record audit (see Results: Step 3 in main manuscript).

Table S1.1. Findings from the audit of admissions for patients with diabetes who had a hypoglycaemia HAC in 2018-19

	**Admissions** n (%)	**Hypoglycaemic events** n (%)
Total n	93	329
Age (years), mean (SD)	66.2 (18.6)	65.5 (18.3)
Gender (male)	45 (48.4)	168 (51.1)
Admission type		
Emergency	72 (77.4)	253 (76.9)
Planned	21 (22.6)	76 (23.1)
Diabetes type		
Type 1	19 (20.4)	70 (21.3)
Type 2	71 (76.3)	246 (74.8)
Gestational	3 (3.2)	13 (4.0)
Hospital division[a]		
Medicine (excluding ED and ICU)	50 (53.8)	145 (44.1)
Surgery	27 (29.0)	117 (35.6)
Obstetrics and gynaecology	9 (9.7)	41 (12.5)
Rehabilitation	7 (7.5)	18 (5.5)
Emergency department (ED)	2 (2.2)	2 (0.6)
Intensive care unit (ICU)	1 (1.1)	1 (0.3)
Dysglycaemia in the 24 hours prior to the event		
Hypoglycaemia (<4.0 mmol/L (72 mg/dL))		119 (36.7)
Hyperglycaemia (>10.0 mmol/L (180 mg/dL))		172 (52.9)
Either		238 (73.2)
Diabetic medications at the time of the event		
Insulin		277 (84.2)
Oral (OHA) or injectable (IHA) hypoglycaemic agents		137 (41.6)
Insulin plus OHA or IHA		122 (37.1)
None		37 (11.2)
Event occurred		
Overnight (between 22.00 and 07.00)		146 (44.4)
Out of hours (outside of Mon-Fri 08.00 to 17.00)		253 (76.9)
Event treated by		
Patient		3 (1.1)
Nurse only		220 (78.0)
Medical officer		49 (17.4)
Medical emergency team (MET) call		10 (3.5)
Contributing causes identified		
Nutrition		179 (54.4)
Poor oral intake (e.g. poor appetite, nausea, vomiting)		115 (35.0)
Fasting		49 (14.9)
Missed or delayed meal		11 (3.3)
Modified diet (e.g. puree)		8 (2.4)
Increased physical activity		25 (7.6)
Diabetic medications		
Insulin increased by 10% or insulin type changed		48 (14.6)
Insulin administration / prescribing error		5 (1.5)
OHA dose increased by more than 10%		11 (3.3)

[a] Missing for 1 patient and 5 events (missing included in percentages). A patient may experience events in multiple divisions.
