# Supplementary material for: A framework for local-level economic evaluation to inform implementation decisions: health service interventions to prevent hospital-acquired hypoglycemia
Source: Int J Technol Assess Health Care. 2023 Dec 20;39(1):e74. doi: 10.1017/S0266462323002775 (PMC11579668; doi:10.1017/S0266462323002775)
Supplement: Gray et al. supplementary material 2 — Gray et al. supplementary material [file S0266462323002775sup002.pdf]

## Supplementary file 2: Literature review templates

The template was used to present a one-page summary of each intervention identified in the literature review to the working group.

Examples of the completed template are provided for the four interventions that were of initially of interest to the working group (see Results: Step 5 in main manuscript).

| | | |
|---|---|---|
| **Study details** | Author (Date) | Mosen et al. (2015)[1] [reports a different analysis of the same intervention / study data as Mularski et al. 2012] |
| | Country | USA |
| | Data years | 2008-11 |
| | Study design | Pre-post |
| | Sample (patients) | Pre: 1277; Post1: 4811; Post2: 5465; Total: 11553 |

| | | |
|---|---|---|
| **Target population** | Setting | Non-ICU; Surgical (Post anesthesia care unit) |
| | Patients | Diabetics, Hyperglycaemics |
| | Target | Glycaemic control, Insulin |
| | Glycaemic target | 70-180 (3.9-10.0) |

| | |
|---|---|
| **Intervention** | Pharmacist service (**Pharmacist led Glycaemic Control Team (GCT)**)<br>Post anesthesia care unit (PACU) for any surgical patient requiring perioperative glycemic control management<br>Pharmacists given training<br>Pharmacists role:<br>- Availability: for consultation 7 days / week [hours?]<br>- Insulin orders and coordinating all aspects of glucose control perioperatively including discharge planning for diabetics<br><br>Evaluated after implementation at 1 year and 2 years |
| | **Pre-intervention** (NR) |

| | | | | | |
|---|---|---|---|---|---|
| **Effect** | **Outcome**<br>Baseline,<br>post-implementation (1y),<br>post-implementation (2y) | **Severe hypoglycaemia** | **Hypoglycaemia**<br><br><70 (<3.9) | **HypERglycaemia** | **Severe hypERglycaemia** |
| | % of patients | | **Significant reduction**<br>pre-post1 RR: 0.38 (pre: 19% v post1: 7.2%)<br>pre-post2 RR: 0.30 (pre: 19% v post2: 5.7%)<br>post1-post2 RR: 0.79 (post1: 7.2% v post2: 5.7%),<br>p: <0.0001 (all) | | |

| | |
|---|---|
| **Audit** | Events in surgical division = 117 (35.6%)<br>Admissions in surgical division ≈ 24 (25.8%)<br>Location of events post-op = 3 (0.9%) [intervention appears to extend beyond immediate post-op period] |
| **Notes** | BGL outcomes evaluated during days 1 to 3 following the PACU admission date |

Effects are formatted as "RR (percentage in baseline v intervention), p value". RR: Relative risk. NR: Not reported. NS: Not significant.

| | | |
|---|---|---|
| **Study details** | Author (Date) | Mularski et al. (2012)[2] [reports a different analysis of the same intervention / study data as Mosen et al. 2015] |
| | Country | USA |
| | Data years | 2008-2010 |
| | Study design | Pre-post |
| | Sample (patients) | Pre: 1294; Post: 4842; Total: 6136 |

| | | |
|---|---|---|
| **Target population** | Setting | Non-ICU; Surgical (**peri-operative**) |
| | Patients | Diabetics, Hyperglycaemics, |
| | Target | Glycaemic control, Insulin |
| | Glycaemic target | 70-180 (3.9-10.0) |

| | |
|---|---|
| **Intervention** | Pharmacist service (**Pharmacist led Glycaemic Control Team (GCT)**)<br>GCT protocol developed by pharmacy clinical coordinator + physicians<br>Pharmacists given education<br>Pharmacists role:<br>- Availability: On call pharmacist, 7 days / week, 10 hours / day<br>- Chart review + Patient meeting (history, patient diabetes education / information, discharge planning) + Nurse meeting<br>- Insulin orders<br>- Daily electronic progress note (structured)<br>Others involved as needed: certified diabetes educator, dietician, and the patient's nurse |
| | **Pre-intervention** (NR) |

| | | | | | |
|---|---|---|---|---|---|
| **Effect** | **Outcome**<br>Pre, during, post | **Severe hypoglycaemia**<br><40 (<2.2) | **Hypoglycaemia**<br><70 (<3.9) | **HypERglycaemia** | **Severe hypERglycaemia** |
| | % of patient days | **Reduction** (p NR)<br>RR: 0.67 (pre: 1.5% v post: 1.0%), p: NR | **Reduction** (p NR)<br>RR: 0.46 (pre: ~10.0% v post: 4.6%), p: NR | | |

| | |
|---|---|
| **Audit** | Events in surgical division = 117 (35.6%)<br>Admissions in surgical division ≈ 24 (25.8%) |
| **Notes** | Surgery = day 1, evaluated BGL outcomes on days 1 to 3 |

Effects are formatted as "RR (percentage in baseline v intervention), p value". RR: Relative risk. NR: Not reported. NS: Not significant.

| | | |
|---|---|---|
| **Study details** | Author (Date) | Rushakoff et al. (2017a)[3]; Rushakoff et al. (2017b)[4] [both papers report on the same analysis of the same intervention / study data] |
| | Country | USA |
| | Data years | 2012-2015 |
| | Study design | Pre-post |
| | Sample (patients) | Pre: 22025; During: 22401; Post: 24079; Total: 68505 |

| | | |
|---|---|---|
| **Target population** | Setting | Hospital-wide; Medical, Surgical, ICU |
| | Patients | All on insulin (insulin pump), Diabetics (type 1), Hypoglycaemics, Hyperglycaemics |
| | Target | Glycaemic control, Insulin, Disglycaemia |
| | Glycaemic target | 70-180 (3.9-10.0) |

| | |
|---|---|
| **Intervention** | **Virtual Glycaemic Management Service (vGMS)** (team includes: endocrinologist, nurse certified diabetes educator (CDE), pharmacist CDE)<br>- EMR based-insulin order sets with BGL measurement orders and hypoglycaemia treatment orders + PoC-BGL automatically uploaded into EMR + carbohydrate intake (grams) entered in EMR<br>- daily automated report (at 05.30) of adult patients with 1+ BGL <70 (3.9 mmol/L) or 2+ BGL ≥225 mg/dL (12.5 mmol/L) in last 24 hours, on insulin pump or type 1 diabetic<br>- vGMS review patients EMR + enter glucose management (insulin) note if required<br>- note viewed by clinicians during morning rounds<br>Evaluation during (implementation year) and post-intervention (year after roll out) |
| | **Pre-intervention:** Had in place an EMR with PoC-BGL measures automatically uploaded in real time, and computerised insuliin orders sets. |

| | | | | | |
|---|---|---|---|---|---|
| **Effect** | **Outcome**<br>Pre, ~~during~~, post | **Severe hypoglycaemia**<br><40 (<2.2) | **Hypoglycaemia**<br><70 (<3.9) | **HypERglycaemia**<br>≥225 (≥12.5) | **Severe hypERglycaemia** |
| | % of patient days | **Significant reduction**<br>RR: 0.31 (pre: 0.033% v post: 0.010%), p: 0.001 | **Significant reduction**<br>RR: 0.63 (pre: 0.78% v post: 0.49%), p: <0.001 | **Significant reduction**<br>RR: 0.61 (pre: 6.6% v post: 4.0%), p: <0.001 | |

| | |
|---|---|
| **Audit** | Scanning hospital-wide (excluding obstetric)<br>Events in reported groups:<br>• Hyper (2x >12.5) within 24 hours = 46 (14.0%)<br>• Hypo (<4.0) within 24 hours = 119 (36.7%)<br>• Type 1 diabetics = 70 (21.3%)<br>• Insulin pump = 0 (from memory 1x during labor with hypo after ceased pump)<br>• All together = 187 (57.7%) |
| **Notes** | Outcome assessed in POC BGLs in days 1 to 28<br>Excluded obstetric patients |

Effects are formatted as “RR (percentage in baseline v intervention), p value”. RR: Relative risk. NR: Not reported. NS: Not significant.

| | | |
|---|---|---|
| **Study details** | Author (Date) | Sinha Gregory, Seley, Ukena et al. (2018)[5] |
| | Country | USA |
| | Data years | 2016-2017 |
| | Study design | Pre-post |
| | Sample (patients) | Pre: 566; Post: 642; Total: 1208 |

| | | |
|---|---|---|
| **Target population** | Setting | Non-ICU; Medical [2x medical units] |
| | Patients | All patients |
| | Target | Insulin, HyPOglycaemia, Hypoglycaemia prevention |
| | Glycaemic target | 70-180 (3.9-10.0) |

| | |
|---|---|
| **Intervention** | **Root cause survey with targeted education**<br><br>**Root cause survey:**<br>1. RN surveys about causes<br>2. Active surveillance - automated electronic tool in EMR (Sunrise) checked for recent hypoglycemic events among the users' patients at login to Sunrise Clinical Manager, if found, launched survey tool on cause of the hypoglycaemia.<br>3. Retrospective chart review of all patients experiencing hypoglycemic events on the two study units<br>4. Responses reviewed / categorised (after 2 months) to identify the top 2 modifiable causes - insulin and changes in nutrition<br><br>**Targeted education:**<br>THEN addressed with targeted education for nurses, physicians, physicians assistants on insulin delivery (10-minute PowerPoint presentation plus one-page handout of main points, focussed on insulin action and a dose adjustment algorithm to titrate insulin). |
| | **Pre-intervention** (NR) |

| | | | | | |
|---|---|---|---|---|---|
| **Effect** | **Outcome** | **Severe hypoglycaemia** | **Hypoglycaemia** <70 (<3.9) | **HypERglycaemia** >180 (>10.0) | **Severe hypERglycaemia** |
| | % of BGL measurements | | **Significant reduction** RR: 0.68 (pre: 2.27% v post: 1.55%), p: <0.001 | **Significant reduction** RR: 0.85 (pre: 38.3% v post: 32.8%), p: <0.001 | |

| | |
|---|---|
| **Audit** | Medical division = 145 (44.1% of events) |
| **Notes** | BGL outcomes during the admission |

Effects are formatted as "RR (percentage in baseline v intervention), p value". RR: Relative risk. NR: Not reported. NS: Not significant.

| | | |
|---|---|---|
| **Study details** | Author (Date) | Aloi et al. (2017)[6] |
| | Country | USA |
| | Data years | NR |
| | Study design | Observational crossover study |
| | Sample (patients) | Total: 993 (x3) crossover trial [crossover study design] |

| | | |
|---|---|---|
| **Target population** | Setting | Non-ICU; Hospital-wide |
| | Patients | All on insulin (insulin pump), Diabetics (type 1), Hypoglycaemics, Hyperglycaemics |
| | Target | Glycaemic control, Insulin [subcutaneous] |
| | Glycaemic target | 140-180 (7.8-10.0) |

| | |
|---|---|
| **Intervention** | **Glucommander** is an **electronic glycaemic management system (eGMS)** that calculated subcutaneous (SC) insulin dosages ('during GM group').<br>- SC insulin initiated by a provider order that calculated total daily dose of insulin as basal and prandial insulin doses.<br>- All daily titrations for basal, prandial and correctional (when needed) insulin doses calculated by eGMS without additional orders from the provider (until the patient was removed from therapy and managed by the provider).<br>- Nurses accessed the eGMS through the EMR.<br>- eGMS recommended full, partial, or held prandial insulin doses through a series of on-screen prompts to the nurse.<br>- Same target glucose range for control (140-180 mg/dL).<br>Evaluation used pre ('before GM'), during GM, and post ('after GM') intervention periods. |
| | **Crossover (control) conditions:** Pre ('before GM') and post ('after GM') intervention periods. SC insulin therapy directed by providers using computerized basal/bolus order set. Initial doses prescribed by body weight in kg or customized at provider's discretion. Titrated daily by provider order as needed. |

| | | | | | |
|---|---|---|---|---|---|
| **Effect** | **Outcome**<br><br>Pre ('before GM')<br>During (GM)<br>Post ('after GM')<br>[crossover trial] | **Severe hypoglycaemia**<br><br><40 (<2.2) | **Hypoglycaemia**<br><br><70 (<3.9) | **HypERglycaemia**<br><br>>180 (>10.0) | **Severe hypERglycaemia** |
| | % of BGL measurements | **No change** (p)<br>pre-during RR: 0.43 (0.14 v 0.06), p: 0.3<br>post-during RR: 0.25 (0.06 v 0.24), p: 0.6 | **Significant reduction**<br>pre-during RR: 0.73 (2.6 v 1.9), p: 0.001<br>post-during RR: 0.68 (1.9 v 2.8), p: 0.001 | **Significant reduction**<br>pre-during RR: 0.71 (51.0 v 36.0), p: 0.001<br>post-during RR: 0.59 (36.0 v 61.0), p: 0.001 | |

| | |
|---|---|
| **Audit** | Hospital-wide = 329 (100%)<br>On insulin = 277 (84.2%) |
| **Notes** | BGL outcomes measured while on each protocol (crossover trial) |

Effects are formatted as "RR (percentage in baseline v intervention), p value". RR: Relative risk. NR: Not reported. NS: Not significant.

**References in Supplementary File 2**


1. Mosen DM, Mularski KS, Mularski RA, et al. Pharmacist glycemic control team associated with improved perioperative glycemic and utilization outcomes. *American Journal of Pharmacy Benefits.* 2015; 7(5): E127-E134.
2. Mularski KS, Yeh CP, Bains JK, et al. Pharmacist glycemic control team improves quality of glycemic control in surgical patients with perioperative dysglycemia. *The Permanente Journal.* 2012; 16(1): 28-33. DOI: 10.7812/tpp/11-131.
3. Rushakoff RJ, Rushakoff JA, Kornberg Z, et al. Remote monitoring and consultation of inpatient populations with diabetes. *Current Diabetes Reports.* 2017; 17(9): 70. DOI: 10.1007/s11892-017-0896-x.
4. Rushakoff RJ, Sullivan MM, MacMaster HW, et al. Association between a virtual glucose management service and glycemic control in hospitalized adult patients: an observational study. *Annals of Internal Medicine.* 2017; 166(9): 621. DOI: 10.7326/M16-1413.
5. Sinha Gregory N, Seley JJ, Ukena J, et al. Decreased rates of inpatient hypoglycemia following implementation of an automated tool in the electronic medical record for identifying root causes. *Journal of Diabetes Science and Technology.* 2018; 12(1): 63-68. DOI: 10.1177/1932296817744808.
6. Aloi J, Bode BW, Ullal J, et al. Comparison of an electronic glycemic management system versus provider-managed subcutaneous basal bolus insulin therapy in the hospital setting. *Journal of Diabetes Science and Technology*. 2017; 11(1): 12-16. DOI: 10.1177/1932296816664746.
