# Supplementary material for: A framework for local-level economic evaluation to inform implementation decisions: health service interventions to prevent hospital-acquired hypoglycemia
Source: Int J Technol Assess Health Care. 2023 Dec 20;39(1):e74. doi: 10.1017/S0266462323002775 (PMC11579668; doi:10.1017/S0266462323002775)
Supplement: Gray et al. supplementary material 3 — Gray et al. supplementary material [file S0266462323002775sup003.pdf]

Supplementary file 3. Estimation of baseline dysglycaemic events

Introduction

Point-of-care blood glucose level (PoC-BGL) test results were used to identify individual hospital-acquired hypoglycaemia and hyperglycaemia events. Plasma glucose test results provided by the pathology labs were available for all patients admitted to both SALHN hospitals, however PoC-BGL test results were only available for Noarlunga Hospital where electronic medical records (EMRs) were in use. On the advice of the working group, only PoC-BGLs were used to identify dysglycaemic events. Assumptions were required to extrapolate the number of dysglycaemic events from Noarlunga data to patients admitted to FMC.

Details of this process are presented in this Supplementary File along with additional details on the codes and definitions used to identify the cohorts of interest.

Data and definitions

Available baseline data

For both hospitals the following data was available for the cohorts:

- Total admissions
- Number of diabetic admissions
- Number of admissions coded as experiencing hypoglycaemia on admission
- Number of admissions coded as experiencing hypoglycaemia during the admission (equivalent to a HAC)
- Number of bed-days for total admissions (i.e. the sum of the length of stay (LOS) for all admissions in the cohort)

In addition, PoC-BGL measures were available for Noarlunga Hospital. Additional information provided for the Noarlunga admissions included:

- Number of admissions with a PoC-BGL measured
- Number of admissions with a PoC-BGL in the specified dysglycaemic ranges
- Total number of PoC-BGL measurements taken
- Number of PoC-BGL measurements within the specified dysglycaemic ranges
- Number of bed-days for admissions with one or more PoC-BGL measured

Definition of the patient cohorts of interest

From local administrative data, summary statistics for total admissions in 2019 for patients aged 18 years or more who met the HAC denominator criteria were obtained. These criteria were defined by the Australian Commission on Safety and Quality in Health Care (ACSQHC).[1]

The HAC denominator criteria excluded admissions for:

- Same-day chemotherapy - DRG V8: R63Z where admission date equalled separation date
- Same-day haemodialysis - DRG V8: L61Z where admission date equalled separation date
- Care type is 'Newborn - unqualified days only'
- Care type is 'Hospital boarder'

- Care type is 'Organ procurement-posthumous'

To match the patient inclusion criteria for the published evaluations of the interventions of interest, two separate cohorts were identified within the FMC data:

(1) All patients, excluding obstetric patients
(2) Surgical patients, excluding obstetric patients

Definition of patients with diabetes (ICD-10 codes)

The following ICD-10 codes (truncated to one letter and two digits) were used to identify patients with **diabetes** within the cohort of interest:

- E10: Type 1 diabetes mellitus
- E11: Type 2 diabetes mellitus
- E13: Other specified diabetes mellitus
- E14: Unspecified diabetes mellitus
- O24: Diabetes mellitus in pregnancy

Definition of hypoglycaemia (ICD-10 codes)

Based on ACSQHC definitions[1] the following ICD-10 codes were used to identify admissions with episodes of **hypoglycaemia** within the cohort of interest:

- E1064: Type 1 diabetes mellitus with hypoglycaemia
- E1164: Type 2 diabetes mellitus with hypoglycaemia
- E1364: Other specified diabetes mellitus with hypoglycaemia
- E1464: Unspecified diabetes mellitus with hypoglycaemia
- E160: Drug-induced hypoglycaemia without coma
- E161: Other hypoglycaemia
- E162: Hypoglycaemia, unspecified

Hypoglycaemia was coded as an **inpatient event** if the condition onset flag indicated it occurred "during admission" (flag equal to 1). Alternatively, hypoglycaemia was coded as occurring or present on admission if the condition onset flag was equal to 2.[2]

Definitions of dysglycaemia

The following definitions of dysglycaemia were used:

- Severe-hypoglycaemia (based on SALHN glycaemia management protocols):
  PoC-BGL <2.2 mmol/L (40 mg/dL) ()
- Hypoglycaemia (based on SALHN glycaemia management protocols):
  PoC-BGL <4.0 mmol/L (72 mg/dL)
- Hyperglycaemia (based on root cause survey intervention):[3]
  PoC-BGL >10.0 mmol/L (180 mg/dL)
- Hyperglycaemia (based on virtual Glycaemic Management Service (vGMS) intervention):[4,5]
  PoC-BGL ≥12.5 mmol/L (225 mg/dL)
- Hyperglycaemia (based on working group recommendations and SALHN glycaemia management protocols):
  PoC-BGL >15.0 (270 mg/dL)

Process for estimating baseline dysglycaemia rates at FMC

The following process was used to estimate the missing PoC-BGL data for the FMC cohort. Table S2.1 provides details of the data available, and indicates the multipliers used for estimation with blue arrows.

1. Estimate the number of admissions with a PoC-BGL measured.

   In the Noarlunga data, the number of admissions with a PoC-BGL measured (1,797) was divided by the number of diabetic admissions (1,443). This gave a multiplier of 1.25. This multiplier was applied to the number of diabetic admissions in the FMC data to estimate the number of admissions with a PoC-BGL measured in the FMC cohort (all patients: 1.25 * 12,018 = 14,966; surgical patients: 1.25 * 3,592 = 4,473).

2. Estimate the number of admissions with a PoC-BGL in the specified dysglycaemic ranges.

   In the Noarlunga data, the number of admissions in each dysglycaemic range was divided by the number of admissions with a PoC-BGL measured (1,797) to give a percentage. This percentage was applied to the number of admissions with a PoC-BGL measured that was estimated for FMC in step 1 above.
   For example, for the all patients cohort the number of admissions with severe-hypoglycaemia was estimated as 0.0083 * 14,966 to give 125 admissions.

3. Estimate the total number of PoC-BGL measurements taken.

   In the Noarlunga data, the total number of PoC-BGL measurements taken (17,817) was divided by the number of admissions with a PoC-BGL measurement (1,797) to calculate the mean number of measurements per separation with a measurement taken (9.91). This multiplier was applied to the FMC estimates obtained in step 1 above.
   For example, for the all patients cohort the total number of PoC-BGL measurements taken was estimated as 9.91 * 14,966 to give 148,389 measurements.

4. Estimate the number of PoC-BGL measurements within the specified dysglycaemic ranges

   In the Noarlunga data, the number of PoC-BGL measurements in each dysglycaemic range was divided by the total number of PoC-BGL measurements (17,817) to give a percentage. This percentage was applied to the total number of PoC-BGL measurements taken that was estimated for FMC in step 3.
   For example, for the all patients cohort the number of PoC-BGL measurements in the severe-hypoglycaemia range was estimated as 0. 0010 * 148,389 to give 150 measurements.

5. Estimate the LOS for admissions with PoC-BGL measurements

   In the Noarlunga data, the LOS for admissions with a PoC-BGL measurement was provided. This number (20,873) was divided by the length of stay for all admissions (35,702) to give a percentage. This percentage was applied to the total LOS for all admissions at FMC.
   For example, for the all patients cohort the LOS for admissions with a PoC-BGL measurement was estimated as 0. 5846 * 279,805 to give 163,587 days.

Table S2.1. Estimated baseline dysglycaemia event rates for FMC during 2019 based on multipliers calculated from Noarlunga data

Cohort	Noarlunga All n	(%)	Multiplier	FMC All (ex. obstetric) n	(%)	FMC Surgical (ex. obstetric) n	(%)	
**Admissions**								
Total admissions[a]	10356			58207		18844		
Diabetic admissions[b]	1443	(13.9)		12018	(20.6)	3592	(19.1)	
Admissions coded as:								
Hypoglycaemia on admission[c]	21	(0.20)		421	(0.72)	54	(0.29)	
Inpatient hypoglycaemia (i.e. a HAC)[c]	4	(0.04)		154	(0.26)	49	(0.26)	
Admissions with PoC-BGL(s) measured	1797		1.25	14966		4473		Estimated[e]
Admissions with PoC-BGL(s) measured divided by diabetic admissions	1.25							
% of non-diabetic admissions with a PoC-BGL measured[d]		(3.97)			(6.38)		(5.78)	
Admissions with PoC-BGL(s) that are:								
<2.2 mmol/L (severe-hypoglycaemia)	15	(0.83)	0.0083	125	(0.83)	37	(0.83)	
<4.0 mmol/L (hypoglycaemia)	77	(4.28)	0.0428	641	(4.28)	192	(4.28)	
>10.0 mmol/L (hyperglycaemia)	463	(25.77)	0.2577	3856	(25.77)	1153	(25.77)	
≥12.5 mmol/L (hyperglycaemia)	306	(17.03)	0.1703	2549	(17.03)	762	(17.03)	
**PoC-BGL measurements**								
Total PoC-BGLs measured	17817		9.91	148389		44351		
Mean number of PoC-BGLs per separation with PoC-BGL(s) measured	9.91							
Number PoC-BGLs that are:								
<2.2 mmol/L (severe-hypoglycaemia)	18	(0.10)	0.0010	150	(0.10)	45	(0.10)	
<4.0 mmol/L (hypoglycaemia)	208	(1.17)	0.0117	1732	(1.17)	518	(1.17)	
>10.0 mmol/L (hyperglycaemia)	5956	(33.43)	0.3343	49604	(33.43)	14826	(33.43)	
≥12.5 mmol/L (hyperglycaemia)	3383	(18.99)	0.1899	28175	(18.99)	8421	(18.99)	
Mean number of PoC-BGLs in this range per separation with PoC-BGLs in this range:								
<2.2 mmol/L (severe-hypoglycaemia)	1.20							
<4.0 mmol/L (hypoglycaemia)	2.70							
>10.0 mmol/L (hyperglycaemia)	12.86							
≥12.5 mmol/L (hyperglycaemia)	11.06							
**LOS (bed-days)**								
LOS for total admissions	35702			279805		72393		
Average LOS for total admissions (days)	3.4			4.8		3.8		
LOS for admissions with one or more PoC-BGL(s) measured	20873	(58.46)	0.5846	163587	(58.46)	42324	(58.46)	Estimated[e]
Average LOS for admissions with one or more PoC-BGLs (days)	11.6			10.9		9.5		

EMR: electronic medical record. HAC: hospital-acquired complication. LOS: length of stay. PoC-BGL: point-of-care blood glucose level. [a] Total admissions in 2019 for patients aged 18 years or more and meeting the HAC denominator criteria (i.e.

excluding admissions for same-day chemotherapy, same-day haemodialysis, or where care type is newborn, hospital boarder, or organ procurement-posthumous). [b] Admissions with ICD-10 codes for diabetes mellitus (type 1 (E10x), type 2 (E11x), other specified (E13x), unspecified (E14x), or in pregnancy (O24x)). [c] Admissions with ICD-10 codes for hypoglycaemia (E1064, E1164, E1364, E1464, E160, E161, E162). If the condition onset flag equalled 1 the event occurred during the patients' hospital admission. If the flag equalled 2 the event did not occur during the admission (i.e. occurred 'on admission'). [d] Assumes all diabetic admissions had a PoC-BGL measurement taken. [e] Estimated values based on those observed at Noarlunga hospital (which used an EMR at the commencement of the project).

Assumptions made during the estimation process

The estimates obtained for FMC are based on the known number of diabetic admissions at both FMC and Noarlunga. The calculation in step 1 assumes that all diabetic patients have at least one PoC-BGL measurement during the admission. The Noarlunga data, suggests that 80% of PoC-BGL tests are in patients with diabetes. This percentage has been applied to the number of FMC patients with diabetes to estimate the number of admissions with one or more PoC-BGL measurements taken. Using this method, a higher percentage of non-diabetic patients at FMC have a PoC-BGL measurement compared to the observed percentage at Noarlunga. This is feasible, given FMC is likely to see a sicker cohort of patients.

The number of admissions coded as inpatient hypoglycaemia or on admission hypoglycaemia was taken directly from the administrative data and no estimation was required. The admissions coded as inpatient hypoglycaemia can be considered to be equivalent to a hypoglycaemia HAC.

The average number of PoC-BGL measurements per separation with at least one PoC-BGL measurement was calculated from the Noarlunga data and used to estimate FMC numbers. This multiplier is the average per separation, and cannot account for differences between diabetic or non-diabetic admissions. It does not take into account LOS effects, for example the average LOS for all admissions at Noarlunga was shorter (3.4 days) than the average LOS at FMC (all patients: 4.8 days, surgical patients: 3.8 days).

References in Supplementary File 3

1. Australian Commission on Safety and Quality in Health Care (ACSQHC). Data from: Hospital-Acquired Complications (HACs) List - Specifications - Version 2.0. 2018. *Sydney, Australia*. Accessed on: 2019 Oct 22. Accessed at: https://safetyandquality.govcms.gov.au/publications-and-resources/resource-library/hospital-acquired-complications-hacs-list-specifications-version-20
2. Australian Institute of Health and Welfare (AIHW). Episode of admitted patient care—condition onset flag, code N. Accessed on: 2021 Aug 3. Accessed at: https://meteor.aihw.gov.au/content/index.phtml/itemId/354816
3. Sinha Gregory N, Seley JJ, Ukena J, et al. Decreased Rates of Inpatient Hypoglycemia Following Implementation of an Automated Tool in the Electronic Medical Record for Identifying Root Causes. *Journal of diabetes science and technology*. Jan 2018;12(1):63-68. doi:10.1177/1932296817744808
4. Rushakoff RJ, Rushakoff JA, Kornberg Z, MacMaster HW, Shah AD. Remote Monitoring and Consultation of Inpatient Populations with Diabetes. *Current diabetes reports*. Sep 2017;17(9):70. doi:10.1007/s11892-017-0896-x
5. Rushakoff RJ, Sullivan MM, MacMaster HW, et al. Association Between a Virtual Glucose Management Service and Glycemic Control in Hospitalized Adult Patients An Observational Study. *Annals of Internal Medicine*. May 2 2017;166(9):621-+. doi:10.7326/M16-1413
