# Supplementary material for: A framework for local-level economic evaluation to inform implementation decisions: health service interventions to prevent hospital-acquired hypoglycemia
Source: Int J Technol Assess Health Care. 2023 Dec 20;39(1):e74. doi: 10.1017/S0266462323002775 (PMC11579668; doi:10.1017/S0266462323002775)
Supplement: Gray et al. supplementary material 4 — Gray et al. supplementary material [file S0266462323002775sup004.pdf]

Supplementary file 4. Detailed methods for the local level economic evaluation (LLEE)

Overview

This supplementary file provides a detailed description of the modelling methods used for the local level economic evaluation (LLEE). It expands on the information provided in 'Step 6. Preliminary LLEE' in the Results section of the main manuscript.

Contents of the Supplementary File:

1. **Process for generating the local dataset:**
   a) Obtained joint distribution of severe-hypoglycaemia and hypoglycaemia events per patient admission from the audit data
   b) Estimated the joint distribution of severe-hypoglycaemia and hypoglycaemia events per patient admission in the FMC cohorts
   c) Generated patient level datasets for the FMC cohorts based on the joint distributions

2. **Process for modelling the intervention effects:**
   a) Published intervention effects (for the preliminary local level economic evaluation)
   b) Elicited intervention effects (for the final local level economic evaluation)
   c) The model
   d) Bootstrapping
   e) Calibration
   f) Modelling scenarios: base case and sensitivity analyses
   g) Reporting criteria for the vGMS intervention
   h) Reported results

1. Process for generating the local dataset

1.a. Obtained the joint distribution of severe-hypoglycaemia and hypoglycaemia events per patient admission from the audit data

To match the patient inclusion criteria for the interventions of interest two cohorts of audit patients were defined: (1) all patients, excluding obstetric patients (n=84) and (2) surgical patients, excluding obstetric patients (n=27). A patient was included in the surgical cohort if any of their hypoglycaemic events occurred in a surgical division. Patients who were coded as having a hypoglycaemic HAC but did not have any recorded BGL measurement <4.0 mmol/L were excluded from the cohorts.

The joint distribution of the number of severe-hypoglycaemia (BGL <2.2 mmol/L) and non-severe hypoglycaemia (BGL ≥2.2 mmol/L and <4.0 mmol/L) events per patient admission was tabulated (Table S4.1). This gave the number of severe-hypoglycaemia events, the total number of hypoglycaemia events (i.e. severe plus non-severe), the number of patients experiencing a severe-hypoglycaemia event, the number of patients experiencing any hypoglycaemia event (i.e. severe or non-severe), and the distribution of these events across the patients in each cohort.

For each cohort the number of patient days on which severe-hypoglycaemia or non-severe hypoglycaemia events occurred was also calculated. Where the patient experienced both severe-

hypoglycaemia and non-severe hypoglycaemia events on the same day, the day was categorised as a severe-hypoglycaemia day.

The audit collected BGL measures for the 24 hours before a hypoglycaemia event, the BGL measure at the time of the event, and two BGL measures after the event. This meant that all hyperglycaemic BGL measures were not collected during the audit and total number of hyperglycaemia events and their distribution could not be estimated. Therefore intervention effects on the rate of hyperglycaemia could not be included in the analyses.

1.b. Estimated the joint distribution of severe-hypoglycaemia and hypoglycaemia events per admission in the FMC cohorts

The distribution of events for the all patients audit cohort was then applied to the all patients FMC cohort using a multiplier. The multiplier was calculated as the total number of hypoglycaemic FMC patients divided by the total number of hypoglycaemic audit patients (641 / 84 = 7.63). The numbers obtained via the multiplier were rounded to the nearest integer, and manually adjusted to ensure the number of events in each severity category matched the total numbers for the FMC cohort (severe-hypoglycaemic patients = 125; total hypoglycaemic patients = 641; severe-hypoglycaemic events = 150; total hypoglycaemic events = 1,732) (Tables S4.1 and S4.2).

The proportion of patients in the surgical cohort for the audit (32.1% of all patients) was very similar to the proportion of surgical patients in the FMC data (30.0% of all patients). Given the small number of audit patients used to derive the surgical cohort distribution (n=27), a more robust approach was to apply the FMC percentage (30.0%) to the event numbers for the FMC all patients cohort generated in the previous step. Once generated minor manual adjustment was used to ensure that the number of events in each severity category matched the total numbers for the FMC cohort (severe-hypoglycaemic patients = 37; total hypoglycaemic patients = 192; severe-hypoglycaemic events = 45; total hypoglycaemic events = 518) (Tables S4.1 and S4.2). These distributions provided information on the number of events and the number of patients experiencing an event.

To calculate the number of hypoglycaemic patient days for each cohort the audit data was examined. In the audit data there was never more than one severe-hypoglycaemia event per day, making the number of severe-hypoglycaemia patient days equal to the number of severe-hypoglycaemia events. In contrast, 16.0% of non-severe hypoglycaemia events occurred on the same day as another event, therefore the total number of hypoglycaemic patient days was equivalent to 84.0% of the total number of non-severe events (225 / 268). These percentages (100% severe-hypoglycaemia events + 84.0% non-severe events) were applied to the numbers obtained in the event distributions above to derive the number of hypoglycaemic patient days per cohort (Table S4.2).

The number of HACs in each cohort was available in the observed data and did not need to be calculated (Table S4.2).

1.c. Generated patient level datasets for the FMC cohorts based on the joint distributions

Two patient-level databases were generated in R [1] based on the manually adjusted FMC distributions. These datasets contained a record for each patient admission, where the patients were assigned a specific number of severe and non-severe events. This created one dataset of 641 patients for the all patients cohort and a dataset of 192 patients for the surgical cohort.

Table S4.1. Joint distribution of severe and non-severe hypoglycaemic events in the patient cohorts

	Severe-hypo events (count per patient)																								
	Audit cohort					FMC all patients cohort (multiplier = 7.63)[a]					FMC all patients cohort (multiplier + adjustment)					FMC surgical cohort (multiplier = 0.30)[b]					FMC surgical cohort (multiplier + adjustment)				
		0	1	2	Total		0	1	2	Total		0	1	2	Total		0	1	2	Total		0	1	2	Total
Non-severe hypo events (count per patient)	0	0	5	0	5	0	0	38	0	38	0	0	37	0	37	0	0	11	0	11	0	0	11	0	11
	1	29	0	0	29	1	221	0	0	221	1	274	0	0	274	1	82	0	0	82	1	81	0	0	81
	2	15	2	0	17	2	114	15	0	129	2	128	19	2	149	2	38	6	1	45	2	39	5	1	45
	3	7	3	0	10	3	53	23	0	76	3	41	29	0	70	3	12	9	0	21	3	13	9	0	22
	4	4	0	0	4	4	31	0	0	31	4	16	0	0	16	4	5	0	0	5	4	5	0	0	5
	5	5	0	0	5	5	38	0	0	38	5	25	0	0	25	5	8	0	0	8	5	8	0	0	8
	6	2	1	0	3	6	15	8	0	23	6	8	5	0	13	6	2	2	0	4	6	2	1	0	3
	7	3	0	0	3	7	23	0	0	23	7	12	0	0	12	7	4	0	0	4	7	4	0	0	4
	8	1	0	0	1	8	8	0	0	8	8	4	0	0	4	8	1	0	0	1	8	1	0	0	1
	9	1	2	1	4	9	8	15	8	31	9	4	7	12	23	9	1	2	4	7	9	1	2	4	7
	11	1	0	1	2	11	8	0	8	16	11	4	0	11	15	11	1	0	3	4	11	1	0	3	4
	29	0	1	0	1	15	0	8	0	8	15	0	3	0	3	15	0	1	0	1	15	0	1	0	1
**Total**					**84**					**642**					**641**					**193**					**192**

Hypo: hypoglycaemia. [a] Multiplier for all patients cohort = total hypoglycaemic FMC patients (n=641) divided by total hypoglycaemic audit patients (n=84) = 7.63. [b] Multiplier for surgical patients cohort = total number of patients in FMC all patients cohort (n=641) divided by total number of patients in FMC surgical patients cohort (n=192) = 0.30.

Table S4.2. Total number of hypoglycaemic patients, events and patient days in the cohorts

		**Audit all patients cohort**	**FMC all patients cohort** (multiplier)	**FMC all patients cohort[a]** (multiplier + adjustment)	**FMC surgical cohort** (multiplier)	**FMC surgical cohort[a]** (multiplier + adjustment)
**Patients**	Severe-hypo	16	123	**125**	39	**37**
	Non-severe hypo	68	519	**516**	154	**155**
	Total hypo	84	642	**641**	193	**192**
**Events**	Severe-hypo	18	139	**150**	47	**45**
	Non-severe hypo	268	1959	**1582**	477	**473**
	Total hypo	286	2098	**1732**	524	**518**
**Patient days**	Severe-hypo[b]	18		**150**		**45**
	Non-severe hypo[c]	225		**1329**		**397**
	Total hypo	243		**1479**		**442**
**HACs[d]**	Total	84		**154**		**49**

FMC: Flinders Medical Centre. HAC: hospital-acquired complication. Hypo: hypoglycaemia. [a] The number of events and patients match the observed numbers for the cohort (for FMC observed is the number estimated from Noarlunga data). [b] Severely-hypoglycaemic patient days are calculated as equal to the number of severe-hypoglycaemia events (based on the audit data). [c] Non-severe-hypoglycaemic patient days are calculated as 0.840 times the number of non-severe hypoglycaemia events (multiplier based on the audit data). [d] Numbers observed for each cohort (no calculation required).

2. Process for modelling the intervention effects

2.a. Published intervention effects (for the preliminary local level economic evaluation)

There were five papers reporting on evaluations of the three interventions of interest. Two interventions were likely to be implemented in the all patients cohort: the root cause survey with targeted education[2] and the virtual Glycaemic Management System (vGMS)[3,4]. While the pharmacist-led peri-operative glycaemic management team (GMT) [5,6] was likely to be implemented only in the surgical patients cohort.

All five papers reported an unadjusted relative risk (RR) for hypoglycaemia. An adjusted odds ratio (OR) for hypoglycaemia was reported by two separate analyses of the same pharmacist-led GMT intervention evaluation data.[5,6] These were converted to RRs using the method described by Zhang et al.[7] Unadjusted RRs for severe-hypoglycaemia were reported for the vGMS [3,4] and pharmacist-led GMT. [5,6] These published RRs were used in the model during the preliminary local level economic evaluation (LLEE) in order to estimate the intervention effect in the specified cohort.

2.b. Elicited intervention effects (for the final local level economic evaluation)

The locally-adjusted RRs elicited during Step 8 of the LLEE framework were used in the model during the final LLEE to estimate the intervention effect in the specified cohort.

2.c. The model

The modelling process is illustrated in Figure S4.1. The numbering below (❶ to ❿) refers to steps illustrated in the figure.

❶ For every event observed in the patient-level database, the probability of that event being prevented was determined by sampling a random number between zero and one. This probability was compared to the RR for the event type (i.e. for a severe or non-severe event). If the probability was greater than the RR, the event was considered to have been prevented. This model conservatively assumed that the intervention independently effected each individual hypoglycaemic event (i.e. preventing one hypoglycaemic event did not affect the probability of preventing a subsequent event in the same patient).

❷ For each patient admission, the number of events that occurred were summed to give the total predicted (modelled) severe, non-severe and total events per patient.

❸ To calculate cohort-level outcomes, the number of events were summed across the cohort for each event type, and the number of patients experiencing each event type were counted. To calculate the predicted (modelled) number of severe, non-severe and total patient days the formula originally applied in the observed data was used (i.e. patient days are equal to 100% of the severe-hypoglycaemia events plus 84.0% of the non-severe events).

Not all patients who experienced a hypoglycaemic event were coded as experiencing a HAC (Table S4.2). The predicted (modelled) proportion of hypoglycaemic patients who were coded as a having a HAC was calculated using the proportions observed in the FMC cohorts at baseline. This was calculated as the number of observed HACs in the FMC cohort divided by the total number of patients who experienced a hypoglycaemic event in the same cohort (24.0% for the all patients cohort; 25.5% for the surgical patients cohort).

❹ The predicted (modelled) percentage of PoC-BGL measurements that were hypoglycaemic (events), patient days that were hypoglycaemic, patients with hypoglycaemia and patients with a HAC were calculated for severe, non-severe and in total for each cohort. The count of patients with one or more PoC-BGL measurements was used as the denominator for patient measures. The total count of bed-days for patients with one or more PoC-BGL measurements was used as the denominator for patient days measures. ❺ The modelled RR was calculated for each outcome as the predicted percentage divided by the observed percentage.

The distribution of severe-hypoglycaemic and hypoglycaemic events per patient was determined for the cohort by cross-tabulating the per patient counts of severe-hypoglycaemic and hypoglycaemic events.

2.d. Bootstrapping

❻ To stabilise the estimates, the model process described above (❶ to ❺ in Figure S4.1) was repeated for 5000 bootstraps. For each bootstrap the seed was changed before sampling the random numbers for the event probability. For each cohort-level outcome, an average value was calculated across the 5000 bootstraps.

2.e. Calibration

The units in which the published and elicited RRs were reported varied for the three interventions of interest (Table S4.3). RRs were calculated from:

- Events (PoC-BGL measurements) in the hypoglycaemic range for the root cause survey intervention [2]
- Patient days with a hypoglycaemic event for the vGMS[3,4]
- Patients with a hypoglycaemic event[5,6] .

The modelled effectiveness analysis needed to apply the RR at the event level, therefore the published and elicited RRs for patient days and patients needed to be calibrated (i.e. converted) in event units.

❾ Calibration began by entering the published (or elicited) RR values (termed the 'published RRs' in the Figure S4.1) into the model as 'input RRs' and running 5000 bootstraps. From the model outputs, 'modelled RRs' were calculated and averaged across the bootstraps, as described in sections 2.c and 2.d above. ❼For each paper, the published RRs were compared to the modelled RRs in the same units (i.e. in events, or patient days or patients). ❽ Where the absolute difference between the published RR and modelled RR was greater than 0.001, the input RR was manually adjusted to reduce the difference. ❾ The revised 'input RR' was then entered into the model and 5000 bootstraps were run. This process was repeated until the modelled RR was within 0.001 units of the published RR.

2.f. Modelling scenarios: base case and sensitivity analyses

Where separate RRs were reported for severe-hypoglycaemia and hypoglycaemia[3-5] the following modelling scenario was applied:

Scenario 1: Separate input RRs for severe-hypoglycaemia and non-severe hypoglycaemia events. The modelled RRs were calibrated to the respective published RRs.

Where a separate RR was not reported for severe-hypoglycaemia[2,5,6] two different modelling scenarios were applied for the interventions. For these interventions scenario 2 was used as the base case (reported in the main manuscript) and scenario 3 for sensitivity analyses:

Scenario 2: Separate input RRs for severe and non-severe hypoglycaemia events. The modelled RRs for both severe and total hypoglycaemia were calibrated to the published RR for hypoglycaemia.

Scenario 3: A common input RR for severe and non-severe hypoglycaemia events. The modelled RR for total hypoglycaemia was calibrated to the published RR for hypoglycaemia.

The input RRs, modelled RRs and comparisons to published RRs are summarised for each modelling scenario in ❿ of Figure S4.1.

2.g. Reporting criteria for the vGMS intervention

One selected intervention involved identifying patients at risk of dysglycaemia for review by a virtual glycaemic management service (vGMS).[3,4] This implied the intervention may be more likely to prevent hypoglycaemia events that occur after an initial dysglycaemia event.

One analysis calibrated the intervention effects across all events in the cohort, while three other analyses calibrated the intervention effects when applied only to events for which patients could have been identified as at risk in the 24 hours prior to the event. Three alternative criteria for identifying at risk patients were specified, based on the criteria reported in the intervention study

and recommendations made by the working group. These were that in the 24 hours prior to the hypoglycaemic event the patient had:

- **Two or more** hyperglycaemic events (PoC-BGL **>15.0 mmol/L** (270 mg/dL)) or one or more hypoglycaemic event(s) (PoC-BGL <4.0 mmol/L (72 mg/dL)) [This criteria is reported in the main manuscript as it was the preferred option of the working group.]
- **One or more** hyperglycaemic events (PoC-BGLs **>15.0 mmol/L** (270 mg/dL)) or one or more hypoglycaemic event(s) (PoC-BGLs <4.0 mmol/L (72 mg/dL))
- **Two or more** hyperglycaemic events (PoC-BGLs **≥12.5 mmol/L** (225 mg/dL)) or one or more hypoglycaemic event(s) (PoC-BGLs <4.0 mmol/L (72 mg/dL)).

For each of the three criteria, the proportion of first and subsequent events that met the criteria were estimated from the clinical audit data (Table S4.4). Each of these proportions were randomly selected in the patient level database. Events not included in the selected proportions were assigned a RR of 1 (i.e. the event could not be prevented by the intervention). Across the 5000 bootstraps the seed was changed before sampling the random numbers used to select the proportions. Modelling scenario 1 was then applied as RRs were reported for both severe-hypoglycaemia and hypoglycaemia.

2.h. Reported results

Results were reported as the average effect across 5000 bootstraps (Main manuscript Table 2; with additional results in Table S4.5). Averaged results were rounded to the lowest whole number to give a conservative estimate (e.g. -47.93 would be rounded to -47). Plots were generated to show the average effect on the joint distributions of severe-hypoglycaemia and hypoglycaemia (Main manuscript: Figure 2; with additional results in Figures S4.2 and S4.3).

Local, patient-level analysis dataset

	Event 1	Event 2	Event 3	...	Event 16	❷ Total events per patient	Baseline	Intervention
**Patient 1**	❶ Non-severe hypo<br>Rand: 0.21<br>< iRR: 0.6760<br>∴ OCCURRED					Severe:<br>Non-severe:<br>Total:	0<br>1<br>1	0<br>1<br>1
**Patient 3**	Severe hypo<br>Rand: 0.58<br>> iRR: 0.3075<br>∴ **PREVENTED**	Non-severe hypo<br>Rand: 0.82<br>> iRR: 0.6760<br>∴ **PREVENTED**	Non-severe hypo<br>Rand: 0.94<br>> iRR: 0.6760<br>∴ **PREVENTED**			Severe:<br>Non-severe:<br>Total:	1<br>2<br>3	0<br>0<br>0
...								
**Patient 641**	Non-severe hypo<br>Rand: 0.12<br>< iRR: 0.6760<br>∴ OCCURRED	Severe hypo<br>Rand: 0.91<br>> iRR: 0.3075<br>∴ **PREVENTED**	Non-severe hypo<br>Rand: 0.61<br>< iRR: 0.6760<br>∴ OCCURRED		Non-severe hypo<br>Rand: 0.89<br>> iRR: 0.6760<br>∴ **PREVENTED**	Severe:<br>Non-severe:<br>Total:	1<br>15<br>16	0<br>14<br>14

		Baseline values: Total numbers	Baseline values: Percentages	Intervention values: ❸ Total numbers	Intervention values: ❹ Percentages	Intervention values: ❺ Modelled RR	Published RRs
**Events**	Severe:<br>Non-severe:<br>Total:	150<br>1,582<br>1,732	0.10<br>1.07<br>1.17	47<br>1,069<br>1,116	0.03<br>0.72<br>0.75	0.31<br>0.68<br>0.64	
**Patient days with an event**	Severe:<br>Non-severe:<br>Total:	150<br>1,329<br>1,479	0.09<br>0.81<br>0.90	47<br>898<br>945	0.03<br>0.55<br>0.58	0.31<br>0.68<br>0.64	0.31<br>NR<br>0.64
**Patients with ≥1 event(s)**	Severe:<br>Non-severe:<br>Total:	125<br>516<br>641	0.84<br>3.45<br>4.28	44<br>466<br>510	0.29<br>3.11<br>3.41	0.35<br>0.90<br>0.80	
**Patients with a coded HAC**	Total:	154	1.03	123	0.82	0.80	

❻ For each bootstrap calculate intervention values (❸ to ❺ above) across all patients.
Run all bootstraps and calculate **average** values across all bootstraps.

❼

❽ **Calibration**: Is the absolute difference between the published RRs and modelled RRs <0.001?

No → Continue calibration by adjusting input RR.

Yes → Calibration complete. Report analysis outputs.

❾ **Input RRs** (iRR). For example: Severe-hypos: 0.3075 Non-severe hypos: 0.6760

❿ Modelling scenarios		Input RRs (i)	Modelled RRs (m)	Published RRs (p)	Calibration comparisons
**Scenario 1**	Severe (S):<br>Non-severe (NS):<br>Total (T):	iS<br>iNS 	mS<br><br>mT	pS<br><br>pT	mS = pS<br>*and*<br>mT = pT
**Scenario 2**	Severe (S):<br>Non-severe (NS):<br>Total (T):	iS<br>iNS 	mS<br><br>mT	 <br>pT	mS = pT<br>*and*<br>mT = pT
**Scenario 3**	Severe (S):<br>Non-severe (NS):<br>Total (T):	iS-NS<br>iS-NS 	 <br>mT	 <br>pT	 <br>mT = pT

Figure S4.1. Process of modelling intervention effects on severe-hypoglycaemia and hypoglycaemia outcomes for the local level economic evaluation (LLEE)

hypo: hypoglycaemia. iRR: input RR. PoC-BGL: point-of-care blood glucose level measurements. RR: relative risk. Rand: Randomly sampled number. S-NS: both severe and non-severe. Process is illustrated for the preliminary LLEE (scenario 1) for the vGMS intervention (with no vGMS reporting criteria applied).

Analysis

The local, patient-level dataset represented severe-hypo and non-severe hypo events (i.e. PoC-BGLs) for every patient in the cohort with a hypo event.

In the local, patient-level dataset:

1. Randomly sampled a number between 0 to 1. Different random values were sampled in each bootstrap run.
   Compared the sampled number [Rand] with the input RR [iRR]. If sampled number > input RR the event was prevented.
2. Summed the number of severe, non-severe and total (any) hypo events for each patient.

Summarised outcomes across all patients:

3. Summed the total number of severe, non-severe and total hypo events.
   Then calculated the total number of:
   - Patients days with a severe, non-severe only, or any hypo event.
   - Patients with severe, non-severe only, or any hypo event(s).
   - Coded hypoglycaemia HACs.
4. Calculated percentages for the step 3 outcomes.
5. Calculated modelled RRs for the step 3 outcomes (as intervention percentage divided by the baseline percentage).

Bootstrapping

6. Steps 3 to 5 were repeated for each bootstrap sample. Outputs from steps 3 to 5 were averaged across all bootstrap samples to give final values.

Calibration

7. Published RRs were compared to modelled RRs (in the same (published) outcome units e.g. patient days in the example).
   Published RRs were in the following units:
   - Events: Root cause survey
   - Patient days: vGMS
   - Patients: Pharmacist-led GMT
8. If the absolute difference between the published RRs and modelled RRs was greater than 0.001, the input RRs were adjusted.
9. The revised input RRs were entered into the model and analysis, bootstrapping and calibration steps were repeated.

Modelling scenarios

10. Three modelling scenarios were applied, dependent on the RRs reported (i.e. total hypo RR only, or separate total hypo RR and severe-hypo RR). RRs used and comparisons made in steps 7 and 8 are illustrated for each scenario.

Table S4.3. Calibration of relative risks for modelling of local intervention effects

	**Published units** (% of …)	**Input RR** (in PoC-BGL units)		**Modelled RR** (in published units)			**Published RR** (in published units)		**Difference in RR** [a]	
		Severe hypo	Hypo	Severe hypo	Non-severe hypo	Hypo	Severe hypo	Hypo	Severe hypo	Hypo
**Step 6: Preliminary analysis (published RRs)**										
**Root cause survey**	PoC-BGLs									
Scenario 2 [b]		0.6760	0.6800	0.68	0.68	0.68	NR	0.68	-0.001	0.000
Scenario 3		0.6800	0.6800	0.68	0.68	0.68	NR	0.68	N/a	0.001
**vGMS** (all Scenario 1)	Patient days									
Criteria: none applied) [b]		0.3075	0.6760	0.31	0.68	0.64	0.31	0.64	0.001	-0.001
Criteria: 2x hyper >15.0 or hypo) [b,c]		0.0000	0.0200	0.50	0.66	0.64	0.31	0.64	0.190	0.000
Criteria: 1x hyper >15.0 or hypo) [c]		0.0000	0.2475	0.41	0.67	0.64	0.31	0.64	0.103	0.000
Criteria: 2x hyper ≥12.5 or hypo) [c]		0.0000	0.2050	0.46	0.66	0.64	0.31	0.64	0.150	0.000
**Pharmacist-led GMT**	Patients									
Scenario 2 – adjusted RR (Mosen) [b]		0.3800	0.2260	0.43	0.43	0.43	NR	0.43	-0.001	0.000
Scenario 3 – adjusted RR (Mosen)		0.2400	0.2400	0.28	0.47	0.43	NR	0.43	N/a	0.001
Scenario 2 – adjusted RR (Mularski) [b]		0.3130	0.1750	0.36	0.36	0.36	NR	0.36	-0.001	0.000
Scenario 3 – adjusted RR (Mularski)		0.1862	0.1862	0.22	0.39	0.36	NR	0.36	N/a	0.000
Scenario 2 – unadjusted RR (Mosen)		0.3325	0.1900	0.38	0.38	0.38	NR	0.38	0.000	0.001
Scenario 3 – unadjusted RR (Mosen)		0.2000	0.2000	0.24	0.41	0.38	NR	0.38	N/a	-0.001
Scenario 1 – unadjusted RR (Mularski)		0.6150	0.2300	0.67	0.41	0.46	0.67	0.46	0.000	-0.001

(Table S4.3 continued…)

**Step 9: Final analysis (locally-adjusted RRs)**										
**Root cause survey** (Scenario 2) [b]	PoC-BGLs									
Most realistic		0.8500	0.8500	0.85	0.85	0.85	0.85	0.85	0.001	0.000
Most optimistic		0.7975	0.8000	0.80	0.80	0.80	0.80	0.80	-0.001	0.000
Most pessimistic		0.9000	0.9000	0.90	0.90	0.90	0.90	0.90	0.000	0.000
**vGMS** (Scenario 1)	Patient days									
Criteria: none applied										
Most realistic		0.4975	0.7900	0.50	0.79	0.76	0.50	0.76	0.001	0.001
Most optimistic		0.2000	0.7575	0.20	0.76	0.70	0.20	0.70	0.001	0.001
Most pessimistic		0.7490	0.8260	0.75	0.83	0.82	0.75	0.82	0.001	-0.001
Criteria: 2x hyper >15.0 or hypo [b]										
Most realistic		0.0010	0.3985	0.50	0.79	0.76	0.50	0.76	0.000	0.000
Most optimistic [c]		0.0000	0.2100	0.50	0.72	0.70	0.20	0.70	0.300	0.000
Most pessimistic		0.4975	0.5100	0.75	0.83	0.82	0.75	0.82	0.000	0.000
Criteria: 1x hyper >15.0 or hypo										
Most realistic		0.1465	0.5250	0.50	0.79	0.76	0.50	0.76	0.000	0.000
Most optimistic [c]		0.0000	0.3975	0.41	0.73	0.70	0.20	0.70	0.213	0.000
Most pessimistic		0.5713	0.6120	0.75	0.83	0.82	0.75	0.82	0.000	0.000
Criteria: 2x hyper ≥12.5 or hypo										
Most realistic		0.0010	0.4000	0.50	0.79	0.76	0.50	0.76	0.000	0.000
Most optimistic [c]		0.0000	0.2125	0.50	0.72	0.70	0.20	0.70	0.300	0.000
Most pessimistic		0.4975	0.5100	0.75	0.83	0.82	0.75	0.82	0.000	0.000

Hyper: hyperglycaemia. Hypo: hypoglycaemia. N/a: not applicable (value not used during calibration). NR: not reported. RR: relative risk. Modelled RR is an average of 5,000 bootstraps. Calibration methods for Scenarios 1 to 3 are described in the methods text (see section 2.e of this supplementary file). [a] Difference is calculated as modelled RR minus published RR. [b] Indicates the base case analysis. [c] The modelled RR for severe-hypoglycaemia could not be calibrated to match the published RR for severe-hypoglycaemia when this criteria was applied. The input RR was set to 0.0000 (i.e. all severe events detected by the criteria were prevented) and total hypoglycaemia events were calibrated.

Table S4.4. Proportion of hypoglycaemic events in the audit data where the patient would have been identified as 'at risk' by the vGMS intervention reporting criteria

	Count of … events			Percentage of … events		
	First	Subsequent	Total	First	Subsequent	Total
**Criteria: none applied**						
Severe hypo	8	10	18			
Non-severe hypo	76	192	268			
Total hypo	84	202	286			
**Criteria: 2x hyper >15.0 or hypo**						
Severe hypo	1	7	8	12.5	70.0	44.4
Non-severe hypo	11	91	102	14.5	47.4	38.1
Total hypo	12	98	110	14.3	48.5	38.5
**Criteria: 1x hyper >15.0 or hypo**						
Severe hypo	3	7	10	37.5	70.0	55.6
Non-severe hypo	18	109	127	23.7	56.8	47.4
Total hypo	21	116	137	25.0	57.4	47.9
**Criteria: 2x hyper ≥12.5 or hypo**						
Severe hypo	2	7	9	25.0	70.0	50.0
Non-severe hypo	17	105	122	22.4	54.7	45.5
Total hypo	19	112	131	22.6	55.4	45.8

Table S4.5. Predicted change in hypoglycaemia occurrence, costs and bed days for interventions of interest to the working group (all analyses, inc. sensitivity analyses)

	PoC-BGLs		Patients				Costs and bed days[a]			
	Severe hypo	Total hypo	Multiple hypos	Severe hypo	Total hypo	Coded HACs[b]	HAC financial penalty (AU$)	Occupied bed days	Occupied bed days costs (AU$)	Nursing time spent on treating hypo events (hours)
**Estimated baseline counts for analysis cohorts**										
All patients	150	1732	330	125	641	154	174,328	1,923	2,788,350	194
Surgical patients	45	518	100	37	192	49	55,468	576	835,200	58
**Step 6: Preliminary analysis (published RRs)**										
**Root cause survey**										
Scenario 2[c]	-48	-554	-89	-34	-115	-27	-30,564	-345	-500,250	-62
Scenario 3	-47	-554	-89	-34	-115	-27	-30,564	-345	-500,250	-62
**vGMS**										
Scenario 1 (criteria: none applied)[c]	-103	-616	-96	-81	-131	-31	-35,092	-393	-569,850	-86
Scenario 1 (criteria: 2x hyper >15.0 or hypo)[c]	-75	-619	-98	-52	-55	-13	-14,716	-165	-239,250	-77
Scenario 1 (criteria: 1x hyper >15.0 or hypo)	-88	-617	-96	-65	-76	-18	-20,376	-228	-330,600	-81
Scenario 1 (criteria: 2x hyper ≥12.5 or hypo)	-81	-618	-97	-58	-71	-17	-19,244	-213	-308,850	-79
**Pharmacist-led GMT**										
Scenario 2 – adjusted RR (Mosen)[c]	-27	-393	-75	-21	-109	-27	-30,564	-327	-474,150	-42
Scenario 3 – adjusted RR (Mosen)	-34	-393	-74	-26	-109	-27	-30,564	-327	-474,150	-44
Scenario 2 – adjusted RR (Mularski)[c]	-30	-421	-81	-23	-122	-31	-35,092	-366	-530,700	-45
Scenario 3 – adjusted RR (Mularski)	-36	-421	-81	-28	-122	-31	-35,092	-366	-530,700	-47
Scenario 2 – unadjusted RR (Mosen)	-30	-413	-79	-22	-118	-30	-33,960	-354	-513,300	-44
Scenario 3 – unadjusted RR (Mosen)	-35	-414	-80	-28	-119	-30	-33,960	-357	-517,650	-46
Scenario 1 – unadjusted RR (Mularski)	-17	-381	-72	-12	-103	-26	-29,432	-309	-448,050	-37

(Table S4.5 continued…)

**Step 9: Final analysis (locally-adjusted RRs)**										
**Root cause survey** (Scenario 2)[c]										
Most realistic	-22	-259	-39	-15	-49	-11	-12,452	-147	-213,150	-29
Most optimistic	-30	-346	-53	-21	-67	-16	-18,112	-201	-291,450	-39
Most pessimistic	-15	-173	-26	-10	-32	-7	-7,924	-96	-139,200	-19
**vGMS** (Scenario 1)										
Criteria: none applied										
Most realistic	-75	-407	-59	-56	-82	-19	-21,508	-246	-356,700	-59
Most optimistic	-120	-503	-72	-96	-105	-25	-28,300	-315	-456,750	-82
Most pessimistic	-37	-312	-47	-26	-61	-14	-15,848	-183	-265,350	-38
Criteria: 2x hyper >15.0 or hypo[c]										
Most realistic	-74	-409	-56	-52	-32	-7	-7,924	-96	-139,200	-59
Most optimistic	-75	-514	-76	-52	-43	-10	-11,320	-129	-187,050	-68
Most pessimistic	-37	-310	-43	-23	-24	-5	-5,660	-72	-104,400	-38
Criteria: 1x hyper >15.0 or hypo										
Most realistic	-75	-409	-57	-54	-47	-11	-12,452	-141	-204,450	-59
Most optimistic	-88	-512	-75	-65	-61	-14	-15,848	-183	-265,350	-72
Most pessimistic	-37	-310	-44	-25	-34	-8	-9,056	-102	-147,900	-38
Criteria: 2x hyper ≥12.5 or hypo										
Most realistic	-80	-486	-71	-58	-53	-12	-13,584	-159	-230,550	-67
Most optimistic	-81	-613	-96	-58	-70	-17	-19,244	-210	-304,500	-78
Most pessimistic	-40	-371	-54	-26	-39	-9	-10,188	-117	-169,650	-44

HAC: hospital-acquired complication. Hyper: hyperglycaemia. Hypo: hypoglycaemia. PoC-BGLs: point of care blood glucose levels. RR: relative risk. Reported change is an average of 5,000 bootstraps. [a] Calculations are described in Results: Step 9 in main manuscript. [b] HACs were calculated in each bootstrap run as a percentage of total hypoglycaemic patients (all patients for root cause survey and vGMS: 24.0%; surgical patients for pharmacist-led GMT: 25.5%). Percentages were derived from FMC baseline data. [c] Indicates base case for each intervention in each analysis.

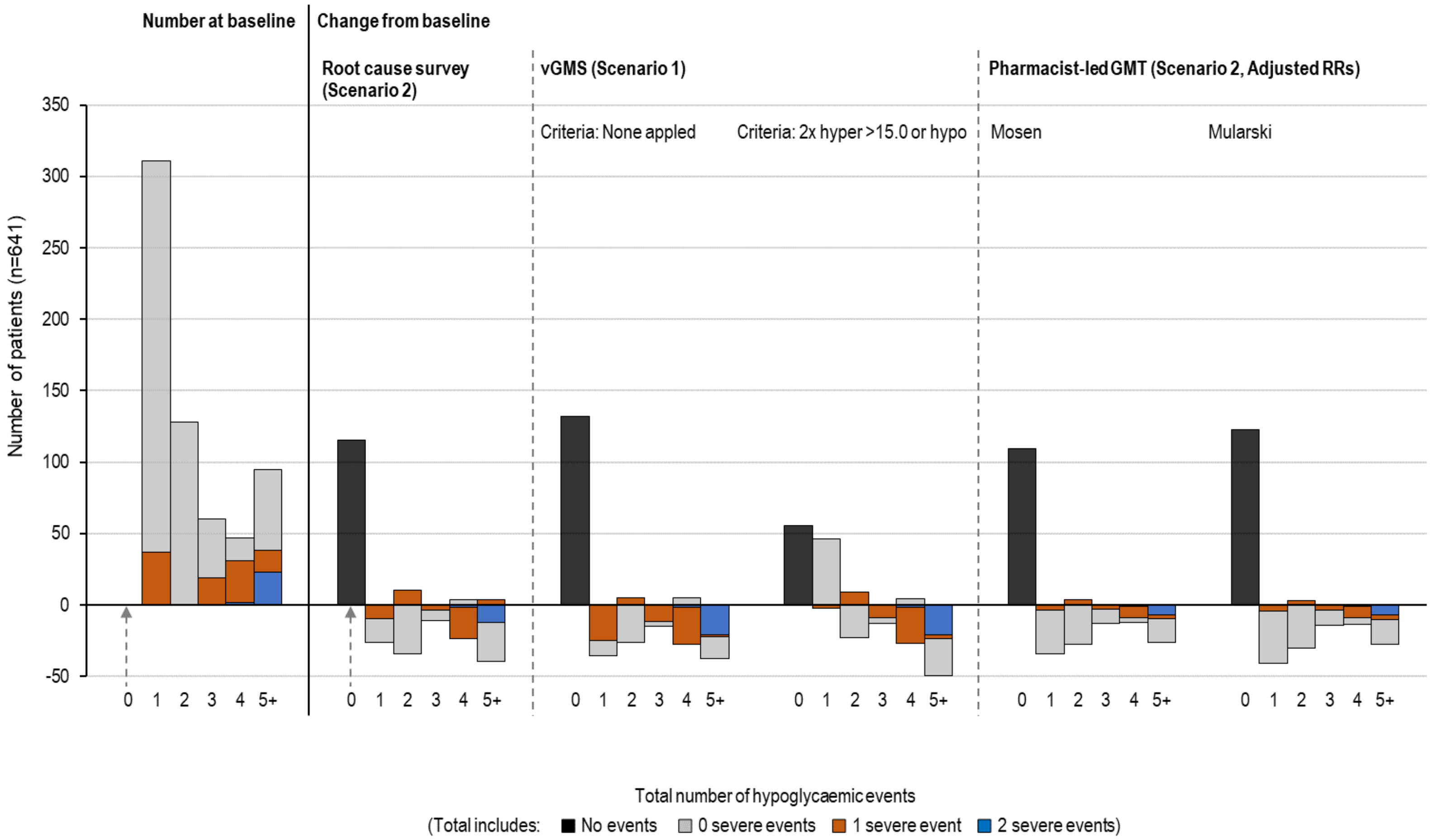

Figure S4.2. Joint distributions of severe-hypoglycaemia and hypoglycaemia events per patient at FMC (base case analyses for **preliminary** local level economic evaluation using published relative risks (RRs))

Estimated baseline and predicted (modelled) post-intervention distributions of hypoglycaemic events across all patients in the FMC cohort who experienced at least one hypoglycaemic event at baseline. Plotted distributions are based on the average intervention effect over 5,000 bootstraps. Distribution for all patients is shown at baseline, while distributions for the interventions show the change in the number of patients experiencing that number / severity of event.

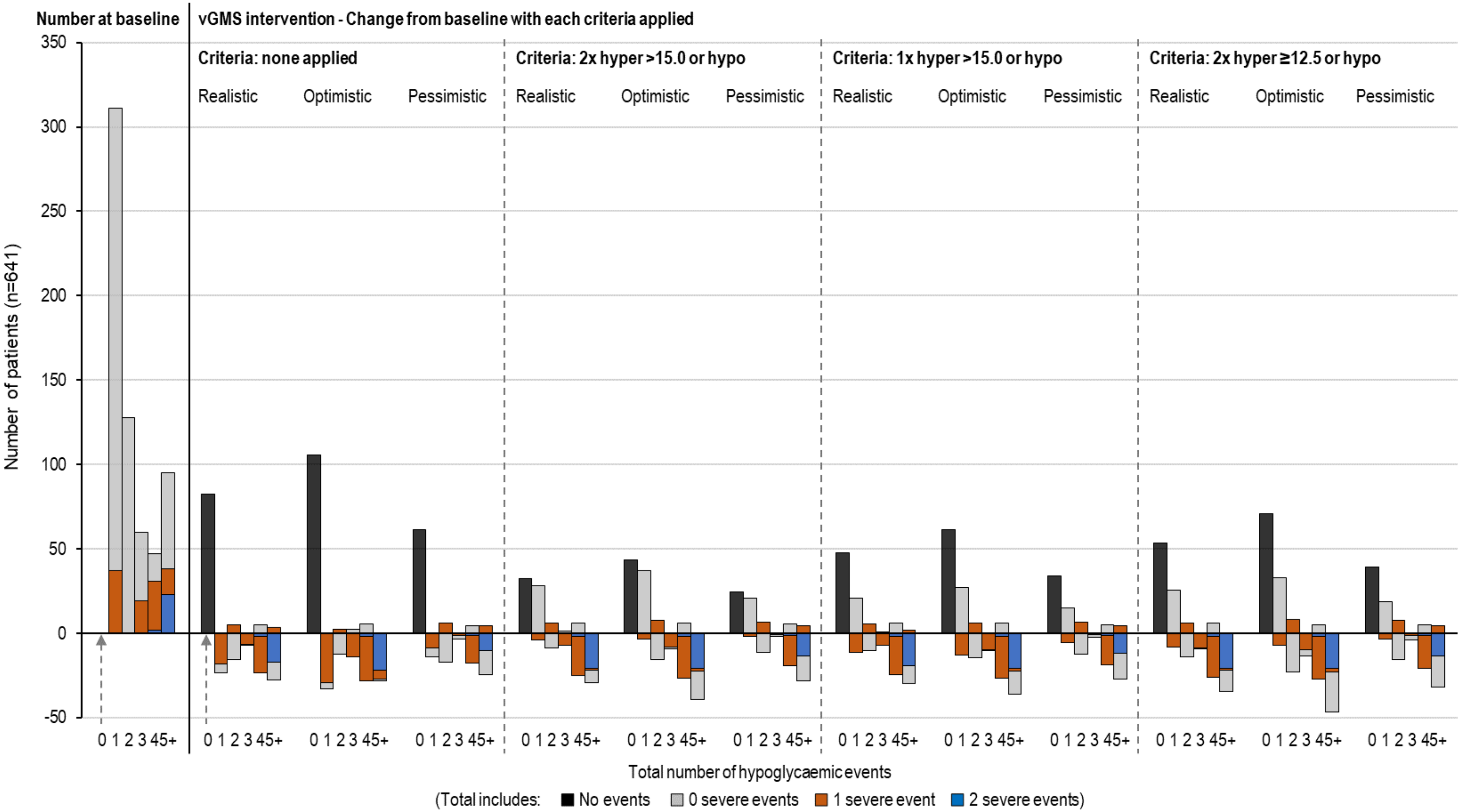

Figure S4.3. Joint distributions of severe-hypoglycaemia and hypoglycaemia events per patient at FMC (vGMS criteria analyses for final local level economic evaluation using locally-adjusted relative risks (RRs))

Estimated baseline and predicted (modelled) post-intervention distributions of hypoglycaemic events across all patients in the FMC cohort who experienced at least one hypoglycaemic event at baseline. Plotted distributions are based on the average intervention effect over 5,000 bootstraps. Distribution for all patients is shown at baseline, while distributions for the interventions show the change in the number of patients experiencing that number / severity of event.

References for Supplementary file 4

1. R Core Team. R version 4.0.2. Vienna, Austria: R Foundation for Statistical Computing; 2020.
2. Sinha Gregory N, Seley JJ, Ukena J, et al. Decreased Rates of Inpatient Hypoglycemia Following Implementation of an Automated Tool in the Electronic Medical Record for Identifying Root Causes. *Journal of diabetes science and technology*. Jan 2018;12(1):63-68. doi:10.1177/1932296817744808
3. Rushakoff RJ, Rushakoff JA, Kornberg Z, MacMaster HW, Shah AD. Remote Monitoring and Consultation of Inpatient Populations with Diabetes. *Current diabetes reports*. Sep 2017;17(9):70. doi:10.1007/s11892-017-0896-x
4. Rushakoff RJ, Sullivan MM, MacMaster HW, et al. Association Between a Virtual Glucose Management Service and Glycemic Control in Hospitalized Adult Patients An Observational Study. *Annals of Internal Medicine*. May 2 2017;166(9):621-+. doi:10.7326/M16-1413
5. Mularski KS, Yeh CP, Bains JK, Mosen DM, Hill AK, Mularski RA. Pharmacist glycemic control team improves quality of glycemic control in surgical patients with perioperative dysglycemia. *The Permanente journal*. Winter 2012;16(1):28-33. doi:10.7812/tpp/11-131
6. Mosen DM, Mularski KS, Mularski RA, Hill AK, Shuster E. Pharmacist Glycemic Control Team Associated With Improved Perioperative Glycemic and Utilization Outcomes. *American Journal of Pharmacy Benefits*. Sep-Oct 2015;7(5):E127-E134.
7. Zhang J, Yu KF. What's the Relative Risk?: A Method of Correcting the Odds Ratio in Cohort Studies of Common Outcomes. *JAMA*. 1998;280(19):1690-1691. doi:10.1001/jama.280.19.1690
